# RIP2 filament formation is required for NOD2 dependent NF-κB signalling

**DOI:** 10.1038/s41467-018-06451-3

**Published:** 2018-10-02

**Authors:** Erika Pellegrini, Ambroise Desfosses, Arndt Wallmann, Wiebke Manuela Schulze, Kristina Rehbein, Philippe Mas, Luca Signor, Stephanie Gaudon, Grasilda Zenkeviciute, Michael Hons, Helene Malet, Irina Gutsche, Carsten Sachse, Guy Schoehn, Hartmut Oschkinat, Stephen Cusack

**Affiliations:** 1 European Molecular Biology Laboratory, 71 Avenue des Martyrs, CS 90181, 38042 Grenoble, Cedex 9 France; 2 Univ. Grenoble Alpes, CNRS, CEA, CNRS, IBS, F-38000 Grenoble, France; 3 Leibniz-Forschungsinstitut für Molekulare Pharmakologie (FMP), Department for NMR-supported Structural, Biology Robert-Rössle-Straße 10, 13125 Berlin, Germany; 4 University Grenoble Alpes, CEA, CNRS, IBS, F-38000 Grenoble, France; 5 European Molecular Biology Laboratory, Structural and Computational Biology Unit, Meyerhofstraße 1, 69117 Heidelberg, Germany; 6 Present Address: Grasilda Zenkeviciute, Department of Pharmacology, University of Cambridge, Tennis Court Road, Cambridge, CB2 1PD United Kingdom

## Abstract

Activation of the innate immune pattern recognition receptor NOD2 by the bacterial muramyl-dipeptide peptidoglycan fragment triggers recruitment of the downstream adaptor kinase RIP2, eventually leading to NF-κB activation and proinflammatory cytokine production. Here we show that full-length RIP2 can form long filaments mediated by its caspase recruitment domain (CARD), in common with other innate immune adaptor proteins. We further show that the NOD2 tandem CARDs bind to one end of the RIP2 CARD filament, suggesting a mechanism for polar filament nucleation by activated NOD2. We combine X-ray crystallography, solid-state NMR and high-resolution cryo-electron microscopy to determine the atomic structure of the helical RIP2 CARD filament, which reveals the intermolecular interactions that stabilize the assembly. Using structure-guided mutagenesis, we demonstrate the importance of RIP2 polymerization for the activation of NF-κB signalling by NOD2. Our results could be of use to develop new pharmacological strategies to treat inflammatory diseases characterised by aberrant NOD2 signalling.

## Abstract (web summary)

Binding of bacterial peptidoglycan muramyl dipeptides induces NOD2 activation and signalling via the downstream adaptor kinase RIP2. Here the authors show that RIP2 forms filaments via its CARD domain, analyse the structure of the CARD filaments and demonstrate the requirement of RIP2 polymerisation for the activation of NF-κB by NOD2.

## Introduction

To respond rapidly to microbial infection, the innate immune system uses pattern recognition receptors (PRRs) to detect pathogen-specific molecules called pathogen-associated molecular patterns (PAMPs)^1^. This primary event leads to receptor activation and signalling via an immediate downstream adaptor protein. Here we focus on the cytosolic PRR NOD2 (nucleotide oligomerization domain 2)^2^ (#285), which senses bacterial infection by recognizing the peptidoglycan breakdown product MDP (muramyl di-peptide)^3,4^, and the adaptor protein RIP2 (receptor-interacting protein 2, also known as RICK^5^.

NOD2 belongs to the Nod-like receptor (NLR) family, which are characterised by three functional domains: a C-terminal ligand-binding domain comprising leucine-rich repeats (LRRs), a central ATP-binding and oligomerization domain (nucleotide oligomerization domain, NOD) and an N-terminal effector death-domain (DD), which in the case of NOD2 is a double CARD (caspase recruitment domain)^6,7^. The downstream adaptor RIP2 belongs to the RIP kinase family and comprises an N-terminal kinase domain, a C-terminal CARD domain and a bridging intermediate domain^8^. Upon cognate ligand binding, NOD2 oligomerizes and recruits RIP2 via CARD-CARD interactions^5,7,9–14^. After RIP2 auto-phosphorylation and ubiquitination, RIP2 becomes a platform for downstream protein effectors including several ubiquitin E3-ligases^15–17^. Eventually, the NOD2 signalling pathway triggers an inflammatory response through NF-κB, MAPK activation and autophagy as well as the production of anti-bacterial peptides, which protect gut epithelial cells from both residual flora and pathogen invasion^7,8,18,19^.

Excessive or absent NOD2–RIP2 signalling is associated with several genetic and non-genetic inflammatory diseases, which lack specific and effective therapies. Loss-of-function single nucleotide polymorphisms (SNPs) in NOD2, which result in impaired epithelial mucosal barrier function, are one of the major genetic susceptibility factors for Crohn’s disease (CD), an increasingly frequent disorder in the western world^20–23^. On the other hand, gain-of-function SNPs of NOD2 can cause Blau syndrome and early-onset sarcoidosis (EOS), which are systematic granulomatous inflammatory diseases^24–27^. Aberrant overactive NOD2-RIP2 signalling might also be involved in inflammatory arthritis, asthma, colorectal cancer and multiple sclerosis, as suggested by animal models and association studies^9^. Despite the importance of the NOD2 signalling pathway in health and disease, there is still an incomplete understanding of its molecular basis. In particular, obtaining insight into the mechanism by which the ligand-induced oligomerization of NOD2 induces RIP2 activation is an important goal since it could lead to the development of new therapies for these clinical conditions.

Recent studies of other intracellular innate immune signalling pathways have shown that ligand-induced PRR oligomerization promotes the polymerization of the adaptor protein through their DDs^28,29^. This interaction leads to the formation of fibrillar protein assemblies, called signalosomes, which link the upstream danger signal to the downstream enzyme-driven pathway. Examples are the anti-viral pathway mediated by RIG-I and downstream adaptor MAVS^30–32^, the inflammasome pathway involving NLRP3, ASC and caspase-1^33,34^, and the recently described T-cell/B-cell signalosome CARMA1-BLC10-MALT1^35^. Several groups have investigated the interaction between NOD2CARDS and RIP2CARD^12,14^, but the question remains whether this hetero-CARD interaction also leads to formation of a higher-order signalosome?

Here we present biophysical and structural data, showing that full-length RIP2 can form filaments that are mediated by RIP2CARD oligomerization. We report the atomic structure of RIP2CARD filaments, solved by high-resolution cryo-electron microscopy (cryo-EM), which reveals the molecular interactions underlying its assembly. We show that NOD2CARDS can bind to one end of the RIP2CARD filaments, suggesting that NOD2 activation could nucleate RIP2 filament formation. Consistent with this, we use structure-guided mutants, designed to specifically disrupt RIP2 filament formation, to demonstrate, in vitro and in cell based assays, the relevance of RIP2 polymerization for the activation of NF-κB signalling following NOD2 stimulation.

## Results

### RIP2 forms filaments in vitro via its CARD domain

**Fig. 1:**
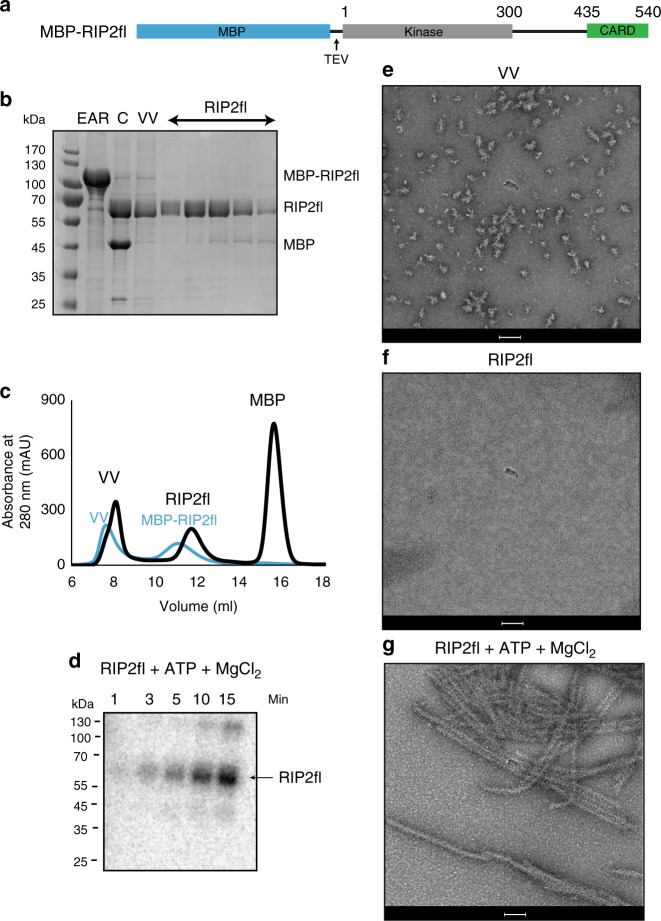
Full-length RIP2 forms filaments in vitro. **a** Domain organization of the MBP-RIP2fl construct used for expression and purification of recombinant RIP2fl from *sf21* insect cells. **b** 12.5 % SDS-PAGE showing recombinant RIP2fl at consecutive purifications steps. **c** Typical size exclusion chromatography profile for tagged (blue) and tag-free RIP2fl (black). **d** In vitro kinase activity of RIP2fl. Complete auto-phosphorylation is achieved after 15 min. **e**–**g** Negative-stain micrographs of (**e**) VV RIP2fl, (**f**) RIP2fl and (**g**) RIP2fl with added ATP and magnesium chloride. Scale bars are 100 nm. EAR: eluate from amylose resin, C: sample after tag cleavage as loaded on size-exclusion chromatography, VV: size exclusion chromatography void volume, Min: minutes

Using the baculovirus system in *sf21* cells, we expressed and purified recombinant full-length human RIP2 with a cleavable maltose-binding protein (MBP) tag at the N-terminus^36^ (MBP-RIP2fl)(Fig. 1a–c). Negative-stain EM of MBP-RIP2fl eluting from the amylose resin shows that the sample is a mixture of aggregates and oligomeric protein (Supplementary Fig. 1a). Addition of ATP and magnesium promotes elongation of the aggregates into a filamentous structure (Supplementary Fig. 1b). After MBP tag cleavage, the protein was further purified by size exclusion chromatography yielding a void volume (VV) fraction and a soluble, non-aggregated fraction denoted RIP2fl (Fig. 1b–c, Supplementary Fig. 2). Analysis of the non-aggregated RIP2fl by electrospray ionization (ESI) mass spectrometry confirmed that the protein is highly phosphorylated (Supplementary Fig. 1c) and an in vitro phosphorylation assay showed that it is capable of further self-phosphorylation and is thus functionally active (Fig. 1d). Negative-stain EM of the VV fraction shows irregular aggregates with some short filaments displaying a central filamentous core (Fig. 1e, Supplementary Fig. 1d-e), whereas imaging the RIP2fl fraction confirms that it is non-aggregated and presumably dimeric (Fig. 1c, f and Supplementary Fig. 2). When ATP and magnesium were added to the non-aggregated RIP2fl fraction, the protein oligomerized into long filaments of diameter 30–40 nm and variable length (0.1–1 μm) and which have a tendency to side-by-side aggregation (Fig. 1g). Extended filaments could also be obtained from the VV fraction by adding non-aggregated RIP2fl, ATP and magnesium, with the VV aggregates acting as seeds (Supplementary Fig. 1f).

**Fig. 2:**
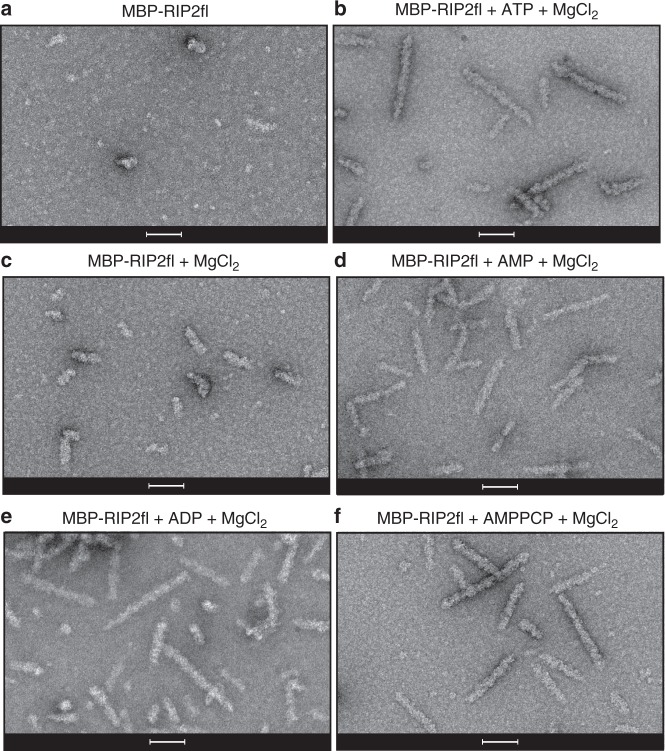
Full-length RIP2 filaments are promoted by nucleotide binding. **a**–**c **Negative-stain micrographs of MBP-RIP2fl filaments obtained from (**a**) MBP-RIP2fl alone, (**b**) MBP-RIP2fl plus ATP dissolved in magnesium chloride buffer, (**c**) MBP-RIP2fl plus magnesium, 5 mM nucleotide dissolved in buffer containing magnesium chloride: (**d**) AMP, (**e**) ADP and (**f**) AMPPCP. Scale bars are 100 nm

We then investigated the importance of different nucleotides in promoting RIP2fl polymerization. For this, we used the uncleaved MBP-RIP2fl fusion protein (Fig. 2a) rather than tag-free RIP2fl. This is because when ATP and magnesium are added, MBP-RIP2fl polymerizes into short filaments (0.1–0.2 μm) that aggregate less compared to those made with tag-free protein, making them easier to visualise by negative-stain EM (Fig. 2b). MBP-RIP2fl polymerization was imaged in the presence of magnesium alone (Fig. 2c) or together with either ATP (Fig. 2b), AMP (Fig. 2d), ADP (Fig. 2e) or the ATP analogue AMPPCP (Fig. 2f). The micrographs reveal that MBP-RIP2fl begins to polymerize in the presence of magnesium and the filaments elongate when any adenosine nucleotide is provided. Given that the recombinant RIP2fl is active and already highly phosphorylated (Fig. 1d and Supplementary Fig. 1c), these data suggest that ATP (or other adenosine nucleotide) boosts RIP2fl polymerization by stabilizing the kinase domain in a pro-filament conformation, rather than by promoting further auto-phosphorylation.

**Fig. 3:**
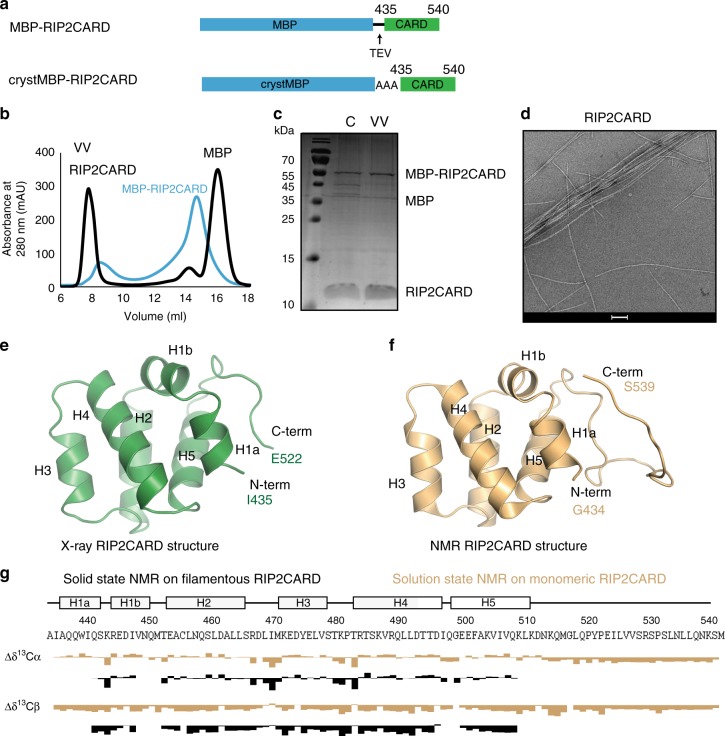
Structure of monomeric RIP2CARD. **a** Domain organization of MBP-RIP2CARD constructs used for expression and purification of recombinant RIP2CARD from *E*. *coli* Rosetta 2. MBP-RIP2CARD was used for characterising the polymerization ability of the CARD domain and for NMR experiments. CrystMBP-RIP2CARD was used for crystallization. **b** Typical size exclusion chromatography profiles of RIP2CARD purification showing both tagged (blue) and tag-free RIP2CARD (black). **c** 17 % SDS-PAGE showing typical sample obtained from RIP2CARD purification. The SDS-PAGE indicates that tag cleavage by TEV is incomplete. **d** Negative-stain electron micrograph of RIP2CARD VV. Scale bar is 100 nm. **e** Ribbon diagram of the RIP2CARD crystal structure reported in this paper **f** Ribbon diagram of the solution NMR structure of RIP2CARD (PDB: 2N7Z). **g** Comparison of secondary chemical shifts of RIP2CARD as a monomer in solution (yellow; BMRB entry: 25828) and in the filament, determined by proton-detected solid-state NMR (black). Experimental ^13^Cα and ^13^Cβ chemical shifts were subtracted from the respective random coil values for each amino acid type (ΔδCα, ΔδCβ)^79,80^. C: sample after tag cleavage as loaded on size-exclusion chromatography, VV: size exclusion chromatography void volume

Interestingly, the polymerized RIP2fl filaments appear to have an inner core decorated with surface projections (Fig. 1g, Supplementary Fig. 1e). In analogy to other adaptor proteins capable of polymerization via their DDs^30,32–34,37^, we hypothesized that the RIP2CARD forms the filament core, whilst the outer projections correspond to the kinase domain. Therefore, we expressed and purified from *E. coli* recombinant RIP2CARD (residues 435–540) with a cleavable MBP tag at the N-terminus (MBP-RIP2CARD, Fig. 3a–c). Upon MBP tag cleavage by the Tobacco Etch Virus (TEV) protease, RIP2CARD mainly migrated in the void volume (VV) of the size-exclusion chromatography column (Fig. 3b). Negative-stain micrographs revealed that RIP2CARD from the VV forms long filaments (Fig. 3d), which have similar length to the RIP2fl filaments, but a smaller diameter of less than 10 nm.

### The structure of monomeric RIP2CARD

To aid structural analysis of the RIP2CARD filaments, we determined the X-ray crystal structure of RIP2CARD (residues 435–540), using a construct with crystallisable MBP^38^ fused at the N-terminus (crystMBP-RIP2CARD, Fig. 3a, e). This construct crystallised in space group *P*2_1_ with four molecules per asymmetric unit (Supplementary Fig. 3). The structure was solved by molecular replacement using NLRP1 CARD domain with a crystallisable MBP at the N-terminus (PDB accession code 4IFP,^39^) as search model and refined at 3.3 Å resolution (Supplementary Table 1). RIP2CARD has the typical CARD fold comprising a Greek key helical bundle with the N- and C-termini oriented in the same direction and with helix H1 broken into two shorter helices: H1a and H1b (Fig. 3e, Supplementary Fig. 3–4). The RIP2CARD crystal structure is very similar to the previously reported solution NMR structure (Fig. 3f, PDB code: 2N7Z)^40^, with a root-mean-square deviation (RMSD) of all Cα positions of 0.95 Å. Interestingly H6 is absent in both the crystal and NMR structures and replaced by a long C-terminal loop, visible only in the NMR structure, which contains putative phosphorylation sites^41–43^.

### The structure of RIP2CARD within filaments

We used solid-state NMR to study the structure of RIP2CARD within the filament. In order to obtain backbone resonance assignments, we recorded ^1^H-detected (H)CANH, (HCO)CA(CO)NH, (HCA)CB(CA)NH, (HCA)CB(CACO)NH, (H)CONH and (H)CO(CA)NH spectra on ^2^H, ^13^C, ^15^N-labeled and 100% back-exchanged RIP2CARD samples at 60 kHz magic angle spinning (MAS)^44^. This data was evaluated together with ^13^C-detected ^13^C-^13^C DARR correlations on protonated samples, that were either uniformly ^13^C-labelled or selectively-labelled using [2-^13^C]- or [1,3-^13^C]-glycerol as carbon source during protein expression^45,46^. The analysis of the ^1^H-detected data yielded the sequence specific assignment for residues Q441 to Q507 (Supplementary Table 2), except for the loop residues 448–451 and 497. The chemical shifts of the assigned residues of filamentous RIP2CARD closely match many chemical shifts of monomeric RIP2CARD in solution showing that the overall conformation is maintained upon filament formation (Fig. 3g). We were not able to assign any cross-peaks to the C-terminal 29 residues that were reported to be flexible by solution NMR investigations^40^. To check whether these signals are absent in our MAS NMR spectra, we inspected ^13^C-^13^C correlation spectra of the samples with a 2- or 1,3-glycerol labelling pattern. At short mixing times, the amino acids Leu, Pro, Thr and Val lead to characteristic cross-peak pattern that allow for a counting of signals. We observed signals corresponding to 9 of 14 leucine residues, 6 of 6 Thr, 5 of 7 Val and only 1 of 4 proline residues (Supplementary Fig. 5). Relying on the distribution of the respective amino acid types in the sequence, this strongly suggests that the missing signals concern residues in the C-terminal segment from 512 to 540. Especially the absence of three proline signal sets, only one being detected, indicates strong structural heterogeneity or mobility in that region where they cluster. Furthermore, the number of missing Leu and Val signal sets corresponds to the number present in the C-terminus and thus corroborates the lack of an ordered structure there, indicating that H6 is also absent in filamentous RIP2CARD.

### NOD2CARDS bind to one end of the RIP2CARD filament

We have shown that both RIP2fl and RIP2CARD samples form filaments in vitro. However, in the cellular context, we expect that such polymerization is initiated by NOD2 oligomerization in response to cognate ligand binding. To recapitulate the core elements of this process, we set out to reconstitute in vitro a filamentous sample comprising the CARDS of both proteins that would be suitable for high-resolution structure determination by cryo-EM.

**Fig. 4:**
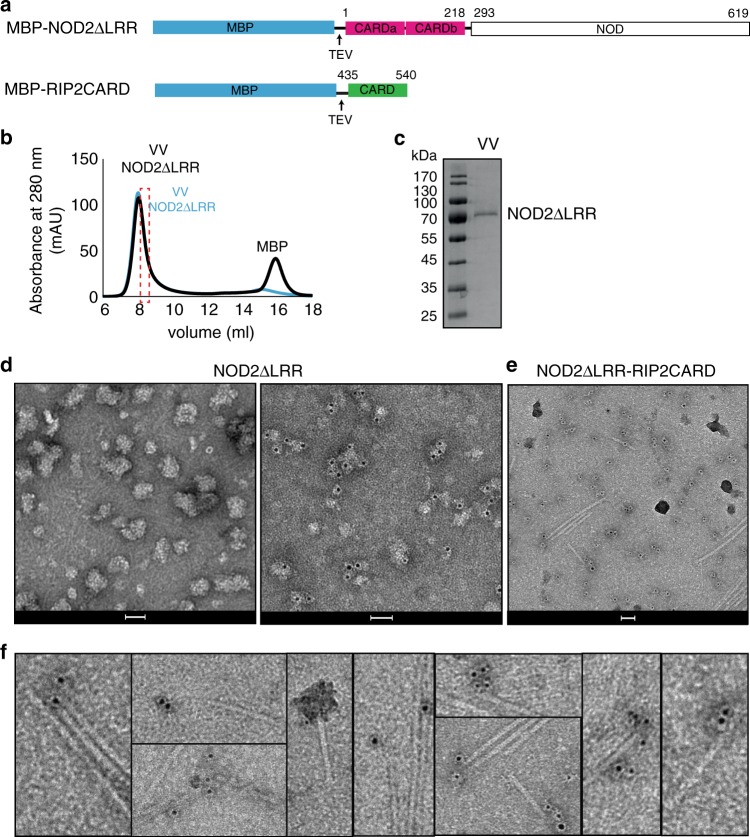
NOD2ΔLRR binds RIP2CARD filaments. **a** Domain organization of NOD2ΔLRR and RIP2CARD constructs used for immuno-gold labelling experiments. Expression of recombinant NOD2ΔLRR was done in *sf21* insect cell. **b**, **c** Size exclusion chromatography profile (**b**) and (**c**) corresponding 12.5% SDS-PAGE showing typical sample obtained from NOD2ΔLRR purification. The size exclusion chromatography profile shows that both tagged (blue) and tag-free NOD2ΔLRR (black) elute in the VV. The dashed rectangular shape indicated the protein fraction used for immuno-gold labelling experiments. **d** Negative-stain images of VV NOD2ΔLRR, corresponding to the fraction used reconstitution with RIP2CARD, unlabelled (left) and after immuno-gold labelling (right). **e**, **f** Example negative-stain micrograph of RIP2CARD filaments with NOD2ΔLRR bound (**e**) and zoom showing gold-particles (black dots) after immuno-gold labelling against NOD2 (**f**). Scale bars are 50 nm. VV: size exclusion chromatography void volume

We first investigated whether NOD2 could be detected by immuno-gold labelling in RIP2CARD filaments formed in the presence of NOD2, following what was previously done for both the AIM2-ASC or NLRP3-ASC complexes^33^. Using the baculovirus insect cell system we expressed and purified a truncated form of NOD2 with a TEV cleavable MBP tag, comprising the CARDS and NOD, but lacking the LRR domain (MBP-NOD2ΔLRR, residues 1–619) (Fig. 4a). This construct is presumed to be derepressed with the CARDS available for interaction. Indeed, a similar NLRP3 construct proved to be a more powerful ASC polymerization promoter compared with the full-length receptor^33^.

Purified and tag-free NOD2ΔLRR eluted mainly in the void volume of a size-exclusion chromatography column (Fig. 4b–c) and consistent with this, negative-stain images showed that NOD2ΔLRR forms soluble aggregates (Fig. 4d). We mixed MBP-RIP2CARD with a less aggregated fraction of NOD2ΔLRR (Fig. 4b, d) and induced filament polymerization by addition of TEV. We then applied immuno-gold labelling against NOD2. As a control, we applied the same immuno-gold labelling to the NOD2ΔLRR sample in the absence of RIP2CARD (Fig. 4d). The control demonstrates that with the protocol used, NOD2ΔLRR can be specifically labelled, although with a heterogeneous number of gold-particles bound per aggregate. Micrographs of the NOD2ΔLRR sample mixed with RIP2CARD showed gold-particles on individual NOD2ΔLRR aggregates or NOD2ΔLRR aggregates bound to RIP2CARD filaments, mostly at one filament-end (Fig. 4e–f).

**Fig. 5:**
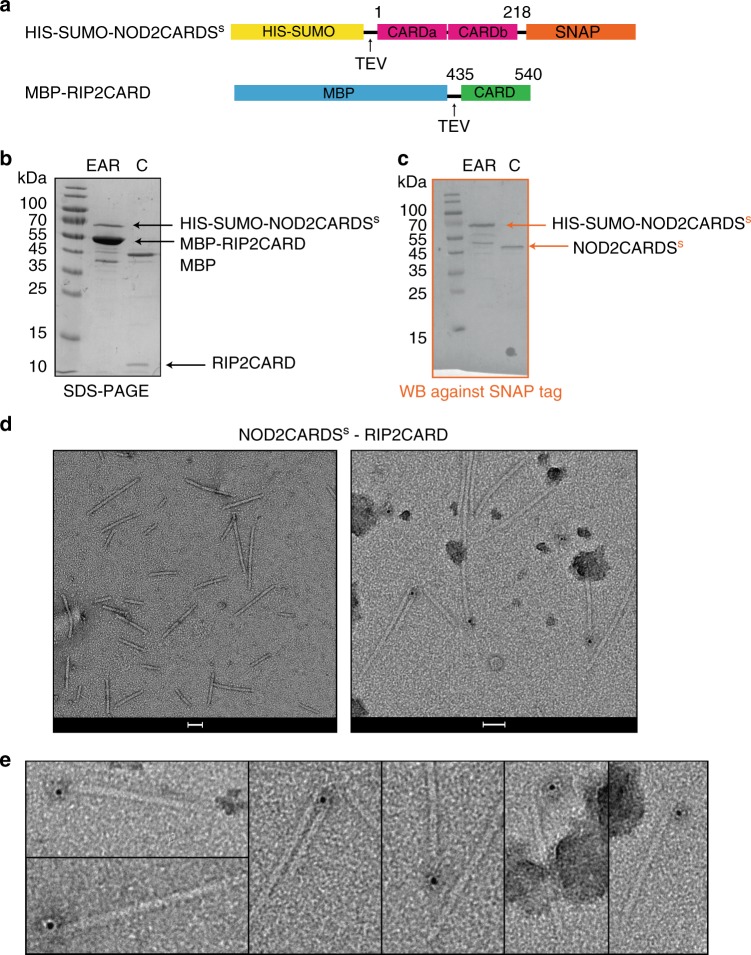
NOD2CARDS bind at one end of the RIP2CARD filaments. **a** Domain organization of HIS-SUMO-NOD2CARDS^s^ and RIP2CARD constructs used for immuno-gold labelling experiments. Expression of recombinant of HIS-SUMO-NOD2CARDS^s^ was carried out in *E*. *coli* Rosetta 2. **b**, **c** 17% SDS-PAGE (**b**) and corresponding WB (**c**) of NOD2CARDS^s^-RIP2CARD co-purification at the amylose elution step (EAR) and after tag cleavage (C) . For clarity, only relevant lanes are labelled. **d** Negative-stain image of RIP2CARD filaments with NOD2CARDS^s^ bound, unlabelled (left) and after immuno-gold labelling (right). Scale bars are 50 nm. **e** Zoom on micrographs of RIP2CARD filaments with NOD2CARDS^s^ bound, showing gold particles (black dots) after immuno-gold labelling against the SNAP tag attached to NOD2CARDS^s^

We then investigated by co-purification and immuno-gold labelling whether the NOD2CARDS are sufficient to interact with the RIP2CARD filament. For this, we used NOD2CARDS (residues 1–218) expressed with a cleavable N-terminal HIS-SUMO tag and a C-terminal SNAP tag (HIS-SUMO-NOD2CARDS^S^) together with cleavable MBP-RIP2CARD (Fig. 5a). Due to the different requirement for optimal expression of these two constructs, we expressed them separately in *E. coli* and then mixed the pellets and co-purified the proteins. After clarification of the crude extract by centrifugation, the supernatant was applied to amylose resin and the eluate was analysed by SDS-PAGE and western blot (WB), using a specific antibody against the SNAP tag (Fig. 5b–c). The results showed that HIS-SUMO-NOD2CARDS^S^ co-elute with MBP-RIP2CARD (Fig. 5b–c). The diameter of filaments observed by negative-stain EM after HIS-MBP tag cleavage was the same as the homo RIP2CARD filaments, while their length ranged from 50 to 500 nm (Fig. 5d).

We then applied immuno-gold labelling against the SNAP tag. The results showed single gold-particles mostly sitting on filament-ends (Fig. 5d–e, Supplementary Fig. 6). In order to evaluate the binding position of NOD2CARDS^S^ on RIP2CARD filament, two more controls were performed with the same immuno-gold labelling protocol: immuno-gold labelling on RIP2CARD filament without NOD2CARDS^S^ (Control 1, C1) and immuno-gold labelling with only the secondary antibody on NOD2CARDS^S^-RIP2CARD filament (Control 2, C2) (Supplementary Fig. 6c, d). Control 1 was used to evaluate the specificity of the primary antibody, whilst Control 2 was used to judge the specificity of secondary antibody. We collected 20 random images on two different grids for each condition at a magnification of ×16,000 and evaluated the number and position of gold-particles in each micrograph (Supplementary Fig. 6f). Final statistics revealed that 70.9% of gold-particles are found on filaments, of which 91.7 % are at one end and we never observe gold-particles at both ends. This shows that NOD2CARDS^S^ are preferentially bound at one end of the RIP2CARD filament. These data are consistent with the hypothesis that under physiological conditions activated NOD2 nucleates RIP2 filament formation yielding a polar assembly.

### Cryo-EM of RIP2CARD filament

To elucidate the architecture of the RIP2CARD filament by cryo-EM, we optimized the protocols for production of both the RIP2CARD and NOD2CARDS^S^-RIP2CARD filaments (Supplementary Fig. 7). For this we used a different RIP2CARD construct encoding for RIP2CARD (residues 431 – 540) with a P3C (human rhinovirus 3C protease) cleavable HIS-MBP tag at the N-terminus (HIS-MBP-RIP2CARD) (Supplementary Fig. 7a). This new RIP2CARD construct dramatically increased the tag cleavage efficiency (compare Fig. 3c with Supplementary Fig. 7c, f). We optimised the purification protocol for NOD2CARDS^S^ -RIP2CARD filaments, by introducing a size exclusion chromatography step before tag cleavage (Supplementary Fig. 7b). This allows aggregates to be discarded and the tagged NOD2CARDS^s^-RIP2CARD complex to be separated from monomeric HIS-MBP-RIP2CARD. NOD2CARDS^s^-RIP2CARD complexes and RIP2CARD were then recombined as described in the Methods. After cleavage, HIS-MBP, HIS-SUMO tags and HIS-tagged proteases, were removed by affinity chromatography followed by dialysis with a high molecular weight cut off (Supplementary Fig. 7c). RIP2CARD filaments were prepared following the same protocol (Supplementary Fig. 7f). Negative-stain and cryo-EM micrographs of samples prepared under the same conditions show that RIP2CARD filaments with bound NOD2CARDS^s^ are shorter, straighter and have a lower tendency to aggregate than RIP2CARD filaments without NOD2 (Supplementary Fig. 7d, e, g, h). Therefore, the hetero-CARD filaments were used for cryo-EM data collection and analysis (Supplementary Table 3).

**Fig. 6:**
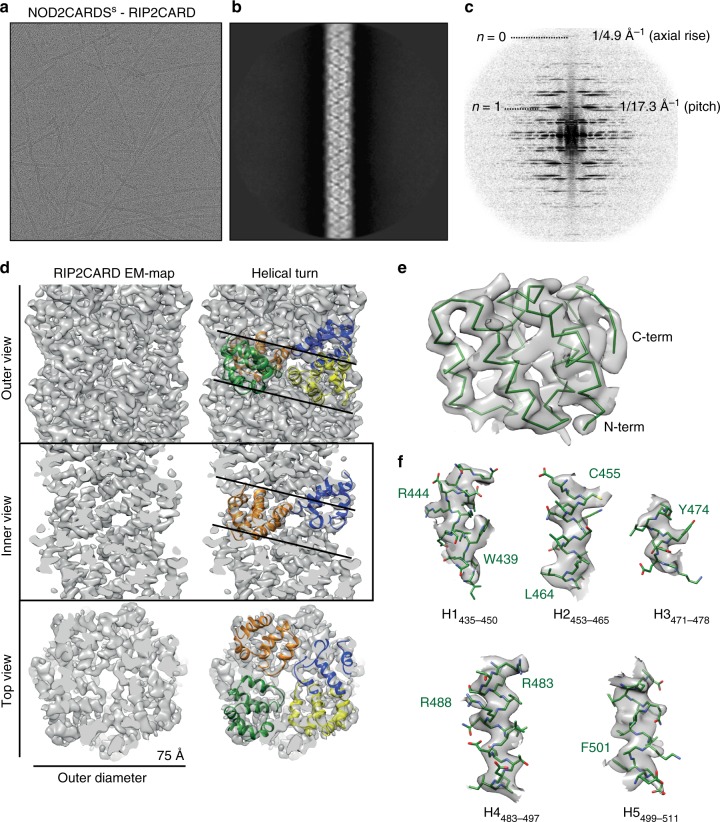
Cryo-EM structure of the RIP2CARD filament. **a** Cryo-EM image of NOD2CARDS^s^-RIP2CARD filaments used for structure determination. **b**, **c** 2D-class average (**b**) and corresponding power spectra (**c**) used for initial symmetry parameter estimation. **d** Final cryo-EM map of the three-dimensional RIP2CARD filament at 3.94 Å resolution (FSC in Supplementary Fig. 8c). Outer, inner and top view without (left) and with (right) RIP2CARD models fitted into one helical turn. **e** View of the RIP2CARD monomer fitted into the EM map showing only the main chain for clarity. **f** Zoomed-in views of the fitting of individual α-helices into the sharpened cryo-EM density. Helices are defined as in the sequence alignment (Supplementary Fig. 4) except for H1, which starts from I435 in the RIP2CARD monomer within the filament. Sidechains are shown as stick models. A few residues with clear EM density for sidechains are labelled

Visual inspection of the individual cryo-EM images, 2D class-averages and corresponding power spectra indicate that the RIP2CARD filament has a helical symmetry (Fig. 6a–c). Indexing of the power spectra and symmetry refinement revealed a left-handed helix of 3.56 subunits/turn with an axial rise of 4.848 Å/subunit (Supplementary Fig. 8a, b). The final cryo-EM map at 3.94 Å resolution (Supplementary Fig. 8c) shows that the filament has an approximate outer diameter of ~75 Å with a central solvent channel of ~25 Å diameter (Fig. 6d). The crystal structure of RIP2CARD can be unambiguously fitted into the cryo-EM density, with both N- and C-terminal ends orientated towards the outside of the filament (Fig. 6e). Manual adjustment using the clear density for the α-helices and the larger side chains such as tryptophan and arginine (Fig. 6f), followed by real space refinement, led to a final atomic model which comprises RIP2CARD residues P433 to Q518 (Supplementary Table 3). This shows that, consistent with the solid-state NMR results, there are only minor structural re-arrangements of the CARD domain upon filament formation (RMSD of 1.12 Å for Cα positions of residues 433–518) and the presumed flexible C-terminus is not observed in the EM map.

### Structure of the RIP2CARD filament

**Fig. 7:**
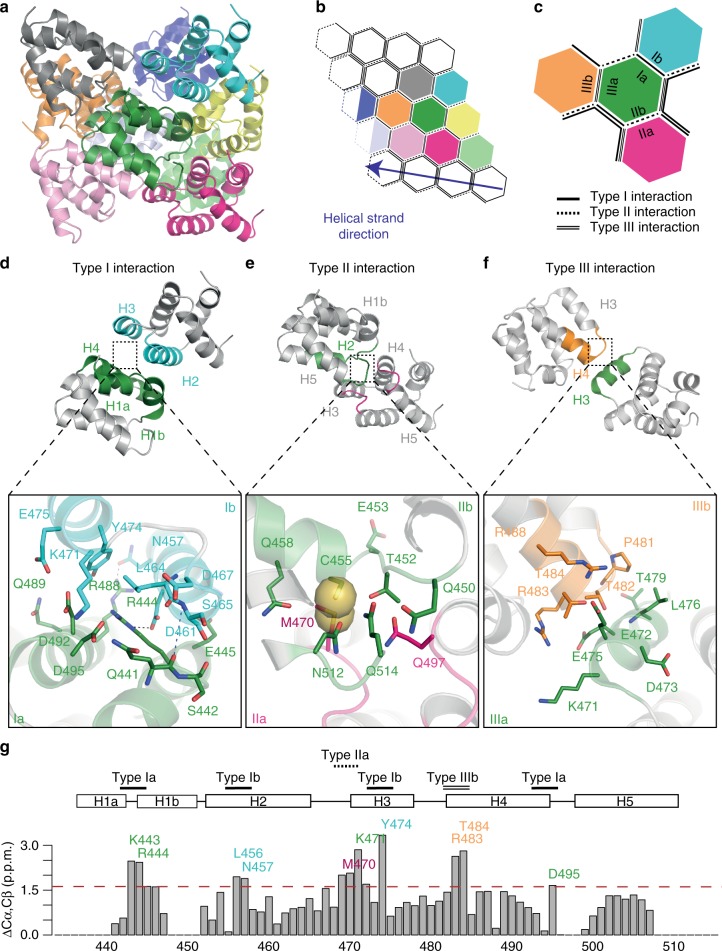
Structural analysis of the RIP2CARD filament assembly. **a** Ribbon diagram of RIP2CARD filament comprising 10 subunits. **b**, **c** Schematic diagram of the helical filament (**b**) and relative orientations of type I, type II and type III interfaces (**c**). Each subunit is represented as a hexagon with the same colour code as in the filament structure in (**a**). Each turn comprises 3.56 subunits. The fourth subunit is represented as a half-empty hexagon to highlight that it is shared with the next turn. Type I, II, III interfaces are represented as a single line, single-dashed line or double line respectively. **d**–**f** Ribbon diagram of RIP2CARD dimers interacting through (**d**) type I, (**e**) type II and (**f**) type III interfaces. Protein regions involved in the interface are highlighted using the colours as in (**c**). The insets show the interactions at relative type surface. H-bonds are represented by black dashed line. Backbone contacts are highlighted by blue dash line, respectively. Sulphurous groups involved in Van der Waals interactions in the type II surface are represented as spheres. **g** Sum of ^13^Cα and ^13^Cβ chemical shift differences between RIP2CARD in the solution and the solid state for each assigned amino acid. Residues that show a strong variation from the mean (1.2 p.p.m.; dotted red line) are labelled in the same colour code as used in (**d**–**f**). The secondary structure elements and positions of the interface surfaces are also shown

The RIP2CARD filament has a similar helical configuration to other CARD filaments already described, such as MAVS CARD, Caspase-1 CARD and the recently described BCL10 CARD filament^30,31,34,35^ (Supplementary Table 4). Following the established convention, the RIP2CARD assembly can be described through interactions at three major asymmetric interfaces, named type I, type II and type III^47^. Type I and II are inter-strand interactions, whilst type III is intra-strand along the helical strand trajectory (Fig. 7a–c).

The type I interface is defined as the interaction between helices H1 and H4 of one molecule (type Ia surface) with H2 and H3 of the adjacent one (type Ib surface). In the RIP2CARD filament, the type I interaction is electrostatic in nature involving several charged residues that form polar interactions (Fig. 7d). D461 and N457 from H3 (type Ib) interact with R444 and R448 from H1 and H4 (type Ia) respectively. The interaction between H1 (type Ia) and H2 (type Ib) is further reinforced by backbone contacts between the break in H1 and the C-terminus of H2 (Fig. 7d, Supplementary Fig. 9a-b). Additional charged or polar residues such as D492, D495, Q441, E445 and Q489 (type Ia) and D467, K471 (type IIb) contribute to the type I interface (Fig. 7d).

The type II interface is normally defined by the interaction between the C-terminal end of H4 and the H4-H5 loop (type IIa surface) and a groove defined by the H1 and H2 corner from one side and H6 helix and its preceding loop on the other (type IIb surface). In the case of the RIP2CARD, which lacks H6, we identified a somewhat different type II interface. The type IIa surface includes the C-terminal end of H4, the H4-H5 and H2-H3 loops, whilst the type IIb surface comprises the H1-H2 loop, the N-terminal of H2 and the visible part of the RIP2CARD C-terminal (Fig. 7e). At the type II interface, the side-chains of M470 (type IIa) and C455 (type IIb) make van der Waals interactions (Fig. 7e and Supplementary Fig. 9c), these side-chains being specific to RIP2CARD (Supplementary Fig. 4). This interface is further reinforced by a polar interaction between N512 and Q458 (Fig. 7e). Moreover, Q497 (type IIa) can interact with the side chains of Q450, T452 and Q514 (type IIb) (Fig. 7e).

The type III interaction normally occurs between H3 (type IIIa) and a groove formed by H1-H2 and the H3-H4 loop (type IIIb). In the RIP2CARD filament, type IIIb comprises the H3-H4 loop and N-terminal of H4, whilst the H1-H2 loop is only contributing to the type IIa surface as described above (Fig. 7f). P481, T482 (type IIIa) and L476 (type IIIb) contribute hydrophobic interactions, whilst E472 and E475 from H3 (type IIIa) potentially form salt bridges with R488 and R483 (type IIIb). Interestingly, both side chain of T482, T484 could swap from the original position in the crystal structure to interact with type IIIa instead of contributing to the intra-αhelical interactions (Fig. 7f).

Figure 7g shows that the most significant chemical-shift differences between RIP2CARD in solution and within the filament, as determined by NMR, map to the subunit interfaces described above. These chemical-shift differences report on local conformational changes due to packing effects in the filament and therefore independently confirm the overall architecture of the intermolecular interfaces observed by cryo-EM. Notably, residues K443, R444, D495, L457, L456, K471 and Y474, located close at the type I interface, undergo strong chemical shift changes in the filament. Residue Y474 shows the largest effect. At its Cγ resonance, multiple contacts in ^13^C-^13^C correlations employing long mixing times are observed, which indicates tight packing of Y474 in the filament. Similarly, conformational changes in the type IIa (M470) and type IIIb (R483, T484) residues lead to significant chemical shift changes of the respective residues, indicating a change in environment of these residues.

### Mutational analysis of the RIP2CARD type II interface

Our immuno-gold labelling results show that NOD2CARDS^S^ bind at one end of the RIP2CARD filament, suggesting that an initial hetero-CARD complex might act as a nucleation point to promote unidirectional RIP2CARD filament growth. Available structures or models of hetero DD complexes, such as RIG-I-MAVS, the Myddosome, the PIDDosome and NLRP3-ASC, show that the DD belonging to the receptor protein continues the helical arrangement of the effector DDs, by forming a combination of the same type I–II-III interfaces. With a view to testing the effect on signalling of site-directed mutants that would uniquely disrupt the RIP2CARD filament structure and not the RIP2-NOD2 hetero-CARD interaction, we modelled the hypothetical hetero-CARD type I, II, III interfaces and evaluated their importance based on the available interaction and mutagenesis studies^12,14^ (Supplementary Fig. 10). As the structure of NOD2CARDS is not yet available and the only reported direct interactions are to the N-terminal NOD2 CARDa (residues 26–122), we computed a three-dimensional NOD2CARDa model using Swiss Modeller^48^ using as template the X-ray structure of the CARD of Nucleolar Protein 3 (PDB code:4UZ0)^49^, which shares 35% identity with NOD2CARDa (Supplementary Fig. 10a), The NOD2CARDa model structure obtained is similar to RIP2CARD (RMSD of 1.70 Å for all Cα positions), consistent with the presumed compatibility of both CARDS to form similar type I-II-III interactions.

NOD2CARDa residues R38, R86, E69, D70 and E72 and RIP2CARD residues E472, D473, E475, D461 and D492 residues have been shown to be important for hetero-CARD interactions^12,14^. Our modelling shows that all these charged residues occur in type I or type III interfaces and are in fact highly conserved between NOD2CARDa and RIP2CARD (Supplementary Fig. 10b-c, and Supplementary Fig. 4). Therefore, mutagenesis of RIP2 residues involved in these interfaces would affect both the interaction with NOD2 and filament formation. Conversely, none of the residues belonging to the observed RIP2 homo-type II interface have been explicitly implicated in the NOD2CARD-RIP2CARD interaction. We deduced that type II interactions within the RIP2CARD filament, notably involving RIP2CARD specific hydrophobic residues C455 and M470, could specifically stabilise homo-interactions within the filament.

**Fig. 8:**
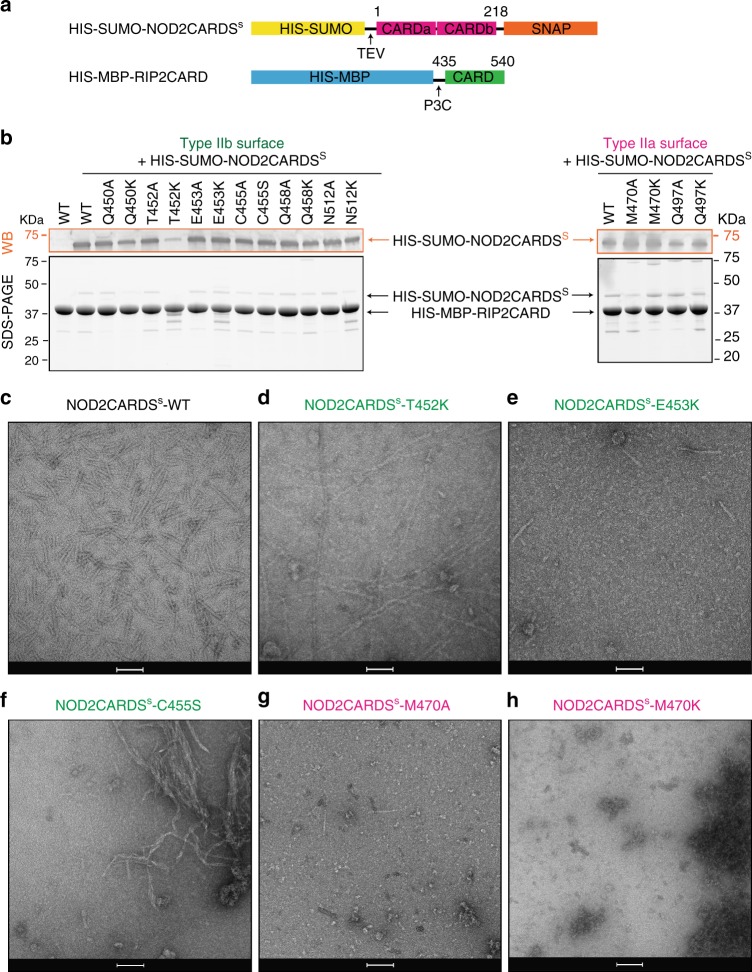
Effect of type II surface mutations on RIP2CARD polymerization. **a** Domain organization of the NOD2CARDS and RIP2CARD constructs used for testing the behaviour of RIP2CARD mutants. **b** Western blot (WB, top panel) and 12.5% SDS-PAGE (bottom panel) of NOD2CARDS^s^-RIP2CARD type II interface mutant co-purification at the amylose elution step (EAR). For clarity, only relevant lanes are labelled. Type IIa and IIb surfaces are coloured as in Fig. 7. **c**–**h** Example micrographs of (**c**) NOD2CARDS^s^-WT, (**d**) NOD2CARDS^s^-T452K, (**e**) NOD2CARDS^s^-E453K, (**f**) NOD2CARDS^s^-C455S, (**g**) NOD2CARDS^s^-M470A and (**h**) NOD2CARDS^s^-M470K, after tag cleavage. Scale bars are 100 nm

We therefore mutated the residues belonging to RIP2CARD type IIa and IIb surfaces (type IIa: M470 and Q497; type IIb: Q450, T452, E453, C455, Q458 and N512) by alanine and lysine substitution (serine for C455). We then assayed the ability of each mutated RIP2CARD construct to bind NOD2CARDS^S^, using the co-purification protocol described above (Fig. 8a, b). Mutant RIP2CARD domains were expressed at similar level to wild-type RIP2CARD and all the mutants displayed unimpaired binding to NOD2CARDS^S^, except for RIP2CARD T452K, which clearly showed lower binding in comparison to wild-type and all the other mutants. We then tested the ability of each construct to polymerize after tag cleavage by imaging the sample with negative-stain EM. The micrographs revealed that many constructs can still polymerise (Supplementary Fig. 11), but with a dramatic change in the filament quality notably for mutants T452K, E453K, C455S, M470A and M470K (Fig. 8c–h). RIP2CARD-T452K forms long filaments but with multiple interruptions compared to wild-type and furthermore they have impaired binding to NOD2CARDS (Fig. 8b–d). This indicates that the mutation negatively affects both binding to NOD2CARDS^S^ and filament quality. Micrographs of RIP2CARD-E453K and RIP2CARD-M470A show particles that might correspond to protein aggregates with rare filaments (Fig. 8e, g). RIP2CARD-C455S forms irregular, more flexible filaments with a high tendency to aggregate (Fig. 8f). M470K micrographs show protein aggregation and absence of filaments (Fig. 8h).

**Fig. 9:**
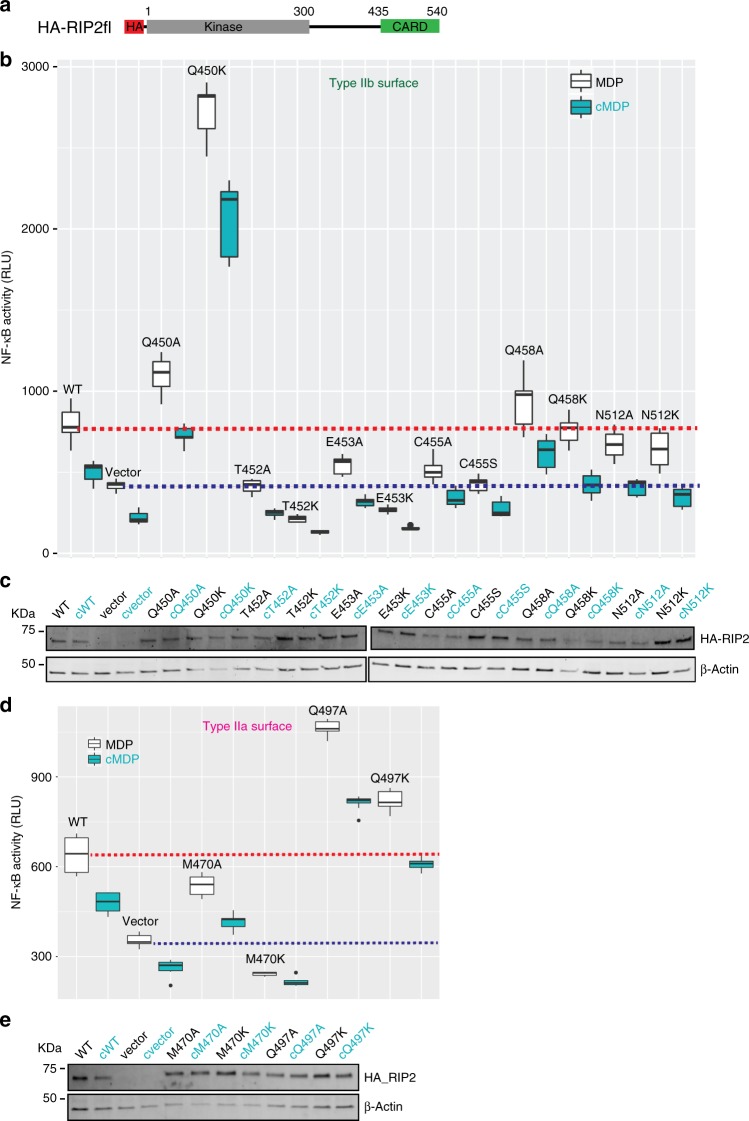
Effect of type II surface mutations on NOD2 dependent NF-κB signalling. **a** Domain organization of the HA-RIP2fl construct used for the luciferase reporter assay. **b**, **d** Luciferase reporter assay in HEK293T cells showing the effect on NF-κB signalling of transfected WT and mutant RIP2fl for (**b**) type IIb surface and (**d**) type IIa surface. Each MDP value is significantly higher than that using **c** MDP. Average RLU values for WT RIP2fl-MDP and control-MDP are shown by dashed red and blue lines, respectively. The boxplot is the graphical representation of the summary statistics of a vector contains information in this order: minimum value (lower whisker), first quartile (25% of data lower bound box), median (50% of data centre line), third quartile (75% of data higher bound box) and maximum value (upper whisker). Black dots on boxplot are extreme values. **c**, **e** Example WB for (**c**) type IIb surface mutants and (**e**) type IIa surface mutants, showing expression of wild-type (WT) HA-RIP2fl and mutants (top) and β-actin (bottom). For each construct, the results for cells induced by either MDP or its control (cMDP, **c**) are reported in black and cyan respectively

We next investigated the effect of these mutations on the activation by NOD2 of transcription factor NF-ĸB, by using a luciferase reporter assay (Fig. 9 and Supplementary Fig. 12). We transiently transfected HEK293T cells with HA tagged RIP2fl mutants (Fig. 9a) together with a plasmid encoding firefly luciferase under the control of NF-ĸB promoter^50^. NF-ĸB activation was induced using the specific NOD2 activator MDP, and cells were lysed 20 h later to record luciferase activity and assay protein expression. In agreement with the in vitro data, mutants Q450, Q458, Q497, N512 show unimpaired NF-κB signalling; with the particularity that Q450K, Q497A and Q497K show high levels of auto-activation compared with the wild-type RIP2 (Fig. 9b, d). In contrast, T452K, E453K, C455S and M470K failed to transmit the signal from NOD2 to NF-κB, as the luciferase value is equal or lower than the control empty vector (Fig. 9b, d). The less drastic alanine mutants T452A, E453A, C455A and M470A also showed lower or zero activity (Fig. 9b, d). All the mutants show similar protein expression to wt RIP2 (Fig. 9c, e and Supplementary Figure 12).

## Discussion

Recent studies on several innate immune systems have shown that recognition of cognate ligands by PRRs that contain a DD (e.g. CARD and PYD) triggers their oligomerization and interaction with the downstream adaptor resulting in the formation of a higher-order filamentous assembly called a signalosome^29,51^. Here we focus on the NOD2-RIP2 signalling pathway, a receptor-adaptor protein combination that shares close structural similarities with PRRs involved in signalosome formation. Specifically, we investigated whether the recruitment of RIP2 by NOD2 via CARD-CARD interactions could lead to the formation of such a signalosome (‘nodosome’). Our biophysical, structural and functional data show that RIP2, via its CARD, can form helical filaments, plausibly nucleated from one end by activated NOD2. Furthermore, we show that RIP2 polymerization is essential for NF-ĸB activation by NOD2, presumably by favouring recruitment of downstream effectors such as the RIP2 ubiquitin ligase XIAP^17^.

The starting point was our finding that phosphorylated and active RIP2fl forms filaments in vitro in the presence of ATP and magnesium. The subsequent observation that RIP2CARD also spontaneously forms more slender filaments, suggests that the CARD domain forms the core of the RIP2fl filaments, while the kinase domain (RIP2K) is on the exterior. Interestingly we observed that not only ATP, but also non-hydrolysable adenosine nucleotides together with magnesium promote polymerization of RIP2fl. This suggests that enhanced RIP2 polymerization by nucleotide-binding results from RIP2K structure stabilization rather than any increase in RIP2K auto-phosphorylation activity. Our previously published biophysical data^36^, show that stable activation of RIP2K involves the coupling of kinase dimerization with auto-phosphorylation of the activation loop. We therefore speculate that CARD polymerization promotes kinase dimerization, by increasing the local RIP2 concentration. Dimerization favours the kinase domain being in the active conformation and therefore able to bind any adenosine-derived nucleotide, independently from the phosphorylation state of the activation loop. In a physiological context, RIP2 could either be already phosphorylated, as it was reported that auto-phosphorylation contributes to protein stabilization^36,52^ or further phosphorylated upon dimerization to stabilize the active conformation. These observations are in line with a recent study showing that an active conformation of the RIP2 kinase, rather than necessarily a catalytically active kinase, is essential for NOD2 signalling, since it permits interaction with the E3 ligase XIAP^17^.

In order to investigate the assembly mechanism of the RIP2 filament core, we co-purified RIP2CARD and NOD2CARDS and used immuno-gold labelling to show that NOD2CARDS bind preferentially at one of the two filament-ends, forming a polar assembly. We then successfully imaged this sample using cryo-EM, obtaining an EM density map at 3.94 Å resolution, where we could unambiguously fit and refine the X-ray structure of RIP2CARD, also reported in this paper. Solid-state NMR confirmed the absence of an ordered structure for the C-terminal segment in the filaments, involving residues beyond K510. RIP2CARD assembles into a left-handed helical filament with 3.56 subunits per turn, a configuration similar to that described for filaments of MAVS, Caspase-1 and, most recently, BCL10 CARDs^30,31,34,35^. RIP2 thus assembles differently from RIP1 and RIP3, which polymerize through their RHIM (RIP homotopic interaction motif) domain into amyloid fibrils^53^.

RIP2CARD assembles into a helical arrangement using the conventional type I, II and III interfaces. Consistent with this, Cα-Cβ chemical shift differences between the solution and solid-state are the most significant for the residues involved at these interfaces (Fig. 3g). The cryo-EM structure reveals that the type I and type III interfaces are polar (Fig. 7d, f), mediated by interactions of R488 and R444 (type Ia surface) with N457 and D461 (type Ib), and by contacts between E472 and E475 (type IIIa surface) and R483, R488 and T484 (type IIIb). The type II interaction is more hydrophobic for instance involving C455 and M470 (Fig. 7e). Y474, which has the highest chemical shift change, is observed buried in the type I interface (Fig. 7d), suggesting that it is not accessible for phosphorylation in the filament. Nonetheless, it is reported that auto-phosphorylation of this residue is important for NOD2 signalling^54^, but might occur at a later stage, after RIP2 ubiquitination by cIAP1^54^.

We then tested the relevance of RIP2 polymerization on the activation of NF-κB by NOD2, by designing RIP2CARD mutants that potentially affect RIP2 polymerization but preserve the interaction with NOD2. Based on available structures of hetero-CARD complexes^31,47^, we expect that a cluster of NOD2CARDS from activated and oligomerized NOD2 could form a short helical extension that preserves the same helical parameters as the RIP2CARD filament. By combining this premise with available mutagenesis data, we deduced that the observed type II interface in the filament, unlike the type I or III interfaces, may be specific to the RIP2 homo-CARD interaction (Supplementary Fig. 10). Therefore, we performed mutagenesis of the RIP2 type II surface with the aim of finding mutants that specifically disrupt homo-, but not hetero-CARD interactions, thus allowing assessing the biological significance of the extended filament. Results from our structure-guided mutagenesis, show that RIP2CARD mutations to lysine (or serine for C455) at the type II interface can affect either hetero- and homo-CARD interactions or only homo-CARD domain interactions. For instance, the T452K mutation both decreased the interaction with NOD2 (Fig. 8b), led to aberrant filament formation (Fig. 8d) and severely affecting signalling (Fig. 9b). On the other hand, mutations of E453, C455 and M470 did not disrupt the interaction with NOD2 but severely affected the quality of RIP2 polymerization and abolished signalling (Fig. 8b, e–h and Fig. 9b, d). The E453 side chain points towards the central channel, suggesting that the lysine mutation disfavours filament elongation by affecting the inner filament electrostatics. Similar reverse charge mutations at the type II interfaces in MAVS CARD filament, combined with others, abrogate MAVS filament elongation^31^. The C455S and M470A/K mutations would reduce the hydrophobic interaction at the RIP2CARD type II interface, consistent with their strong effect on the quality of filaments or whether filaments form at all (Fig. 8f–h). Interestingly, some alanine mutants (e.g. T452A, E453A and C455A) also show a significant decrease in the NF-κB signal, despite the fact that they do not affect the ability of RIP2CARD either to bind NOD2CARDS or to polymerize in vitro. We speculate that alanine mutations have a milder effect, compared to changing the charge, that cannot be detected by negative-stain EM, except for the case of M470A, whose severe disruptive affect highlights the importance of this RIP2 specific residue in forming the type II interface in the filament.

**Fig. 10:**
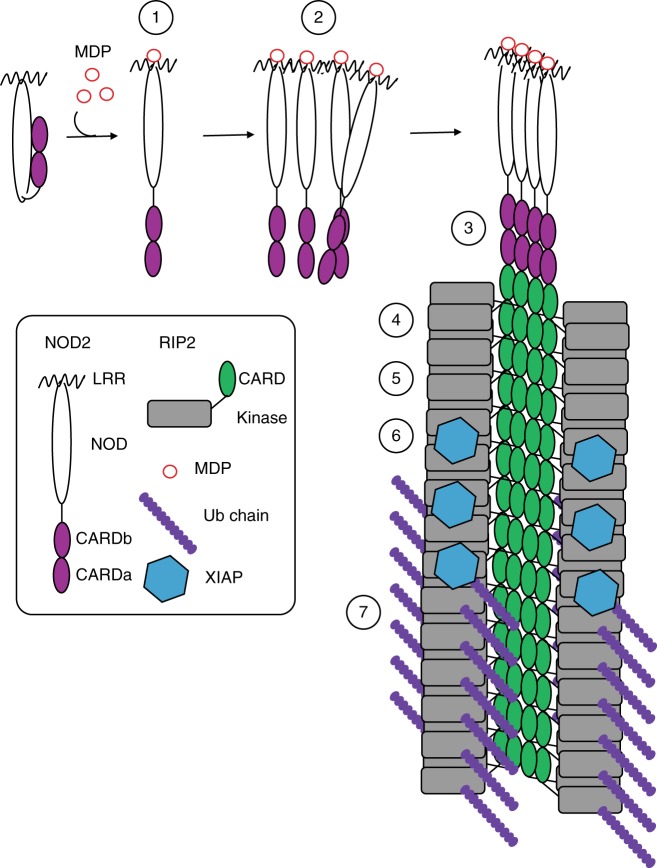
Model of nodosome assembly. Model of nodosome assembly, based on the results described here, other published data and analogy to other signalosome systems. Steps 1–7 in the process are discussed in the main text. The legend for the components of the system is in the inset

In conclusion, we provide evidence for the existence and biological importance of a NOD2-RIP2 polar filamentous assembly, which is likely the core of the nodosome complex. Based on the results described here, other published data and analogy to other signalosome systems, we propose that nodosome assembly occurs as follows (Fig. 10): (1) binding of MDP to NOD2 LRR domain activates the receptor causing derepression of the CARDs; (2) NOD2 oligomerises via its NOD and CARD domains; (3) oligomerized NOD2 recruits RIP2 via its CARD domain forming the hetero-CARD complex (4) cumulative binding of RIP2 to the hetero-CARD complex promotes filament elongation to form the helical assembly here described; (5) polymerization of RIP2CARD in the presence of ATP stabilises the active antiparallel dimeric form of RIP2K; (6) E3 ligases, such as XIAP bind the active from of RIP2K^17^; (7) RIP2 becomes K63-ubiquitinated enabling it to recruit downstream effector proteins.

Finally, by combining our biophysical, structural and functional analysis with existing data, we provide essential information that could potentially be used to explore new therapeutic options for inflammatory diseases characterised by aberrant NOD2-RIP2 signalling.

## Methods

### Constructs, protein expression and purification

Constructs described in this paper were generated from pcDNA3 plasmids encoding human RIP2 and human NOD2^50^. The N-terminally HIS-tagged TEV (Tobacco Etch Virus) and P3C (human rhinovirus 3C) proteases used in this paper were produced at the Protein Expression and Purification Core Facility at EMBL, Heidelberg, Germany.

Recombinant human RIP2fl and human NOD2ΔLRR(1–619) were produced using the baculovirus system in *sf21* insect cells. The DNA sequence of RIP2fl and NOD2ΔLRR were cloned from pcDNA3 into the vector pFastBacHTB using NcoI and HindIII cloning sites. Using the *In-Fusion* cloning technology (Takara Clontech), the original TEV cleavable HIS tag was replaced with a TEV cleavable maltose-binding protein (MBP) tag, which improved both expression and stability of recombinant proteins during insect cells expression (MBP-RIP2fl and MBP-NOD2ΔLRR). MBP-RIP2fl and MBP-NOD2ΔLRR were expressed and purified following the same protocol^36^.Virus generation and amplification, insect cell infection and protein expression were performed at the EMBL Eukaryotic Expression Facility. Briefly, Sf21 cells at 0.6 × 10^6^ cells ml^−1^ were infected with a virus shot able to stop cells growing in 24 h. On the fourth day post-infection, cells were harvested and re-suspended in 1 × 10^−1^ (v/v) ratio of lysis buffer 20 mM Tris pH 7.5, 50 mM NaCl, 2 mM β-mercapto-ethanol (βMe), 0.01% NP40 supplied with protease cocktail inhibitor (Complete, Roche). Using a douncer, cells were homogenized and afterwards centrifuged at 18,000 × *g* for 30 min. The resulting supernatant solution was incubated for at least 2 h with amylose-affinity chromatography resin (New England Biolabs), whilst gently shaking at 4 °C. The fusion protein was then eluted using the same lysis buffer supplemented with 40 mM maltose. Upon overnight TEV cleavage, either RIP2fl or NOD2ΔLRR were applied to size exclusion chromatography, using a similar buffer composition of lysis buffer (20 mM Tris pH 7.5, 50 mM NaCl, 0.5 mM TCEP).

For crystallization purposes, RIP2CARD (435–540), was cloned into pETXM1 plasmid using the NcoI and XhoI cloning sites, resulting in a protein construct with an N-terminal crystallisable MBP (crystMBP-RIP2CARD) spaced by a three alanine linker. The construct was expressed in *E. coli* Rosetta 2 (Novagen) by growing the bacterial culture at 37 °C until an OD_600 nm_ of 0.6 and inducing with 0.3 mM IPTG (isopropyl-β-D-1-thiogalactopyranoside) overnight at 16 °C. The cells where harvested, re-suspended in 1 10^−1^ (v/v) ratio of lysis buffer 20 mM Tris pH8, 50 mM NaCl, 2 mM (β-Me) containing protease cocktail inhibitor (Complete, Roche) and lysed by sonication. The crude extract was centrifuged for 30 min at 18,000 × *g* and the soluble fraction was applied to amylose-affinity chromatography resin (New England Biolabs) and purified as described above. After elution, the fusion protein was applied to a prepacked anion exchange chromatography column (GE Healthcare) with a 0 to 1 M NaCl gradient. The protein was further purified on a Superdex 200 size exclusion chromatography column (GE Healthcare), in buffer containing 20 mM Tris pH8 and 50 mM NaCl. The protein corresponding to the monomeric peak was used immediately for crystallization purposes.

For immuno-gold labelling experiments and solid state NMR, RIP2CARD (435–540) was cloned in pETM40, which results in RIP2CARD with N-terminal MBP tag (MBP-RIP2CARD). Protein was expressed in *E. coli* Rosetta 2 by growing the bacterial culture at 37 °C until OD_600nm_ of 0.6 and inducing with 0.3 mM IPTG for 4 h at 18 °C. Protein was either purified as described for crystMBP-RIP2CARD, or combined with NOD2CARDS^s^ as described in the immuno-gold labelling section.

For production of the NOD2CARDS^s^-RIP2CARD filament sample, RIP2CARD (431–540) was cloned into pETM41 using the *in-Fusion* cloning technology, which resulted in a fusion protein with N-terminal HIS-MBP tag (HIS-MBP-RIP2CARD). To improve the cleavage efficiency, the original TEV site was replaced by a P3C cleavage site. Protein was expressed as described for MBP-RIP2CARD except that media was supplied with 0.04% (w/v) glucose to reduce bacterial MBP expression. The cells were harvested and either used for RIP2CARD filament production or combined with bacterial pellets containing HIS-SUMO- NOD2CARDS^s^ protein to prepare NOD2CARDS^s^ -RIP2CARD filament sample.

NOD2CARDS (1–218) was cloned in pETM15 using the restrictions sites NcoI and XhoI. By applying the technology *in-Fusion*, a TEV cleavable HIS-SUMO tag was added to the N-terminus, while a non-cleavable SNAP tag was added to the C-terminus (HIS-SUMO-NOD2CARDS^s^). The construct was transformed and expressed in *E.coli* Rosetta 2 by growing the bacterial culture supplied with 0.04% (w/v) glucose at 37 °C until an OD_600 nm_ of 0.6 and inducing with 0.3 mM IPTG overnight at 16 °C.

For production of RIP2CARD filaments bound to NOD2CARDS^s^, 1 L of RIP2CARD culture (or ~5 g of bacterial pellet) was combined with 100 ml of NOD2CARDS^s^ (or ~0.9 g of bacterial pellet) and resuspended in 100 ml of lysis buffer (20 mM Tris pH 8, 50 mM NaCl, 1 mM TCEP and Complete proteases inhibitor cocktail (Roche)). The sample was then lysed by sonication and crude extract was let on gentle shacking for 30 min at 4 °C. After centrifugation at 18,000 × g for 30 min, the soluble fraction was incubated for 2 h with amylose-affinity chromatography resin. The sample was then eluted using lysis buffer supplemented with 40 mM maltose and complex formation was checked on SDS-PAGE. The amylose eluate was successively passed through size exclusion chromatography column Superose6 (GE Healthcare) equilibrated in 20 mM Tris pH 8, 50 mM NaCl, 1 mM TCEP to separate aggregates, NOD2CARDS^s^-RIP2CARD complex and RIP2CARD monomer. NOD2CARDS^s^-RIP2CARD complex at 0.25-0.35 mg ml^−1^ and monomeric RIP2CARD at 0.45–0.55 mg ml^−1^ were then combined at the desired ratio (usually 1 × 10^−1^ v/v) and filament formation was induced by adding protease overnight at 4 °C. HIS-MBP and HIS-SUMO tags, proteases and uncleaved protein were removed by applying the sample to a Ni-NTA resine (Takara). Further purification of filaments was achieved by dialysis overnight using a membrane with a 300 kDa cut off. SDS-PAGE and negative-stain EM were used along the purification procedure to check sample homogeneity and filaments formation. Filaments containing only RIP2CARD were obtained and purified following a similar protocol. To avoid CARD protein aggregation all the purification steps were carried out at 4 °C.

### Gel filtration calibration curve

The calibration curve of the Superdex 200 column (GE Healthcare) used to purify RIP2fl, RIP2CARD and NOD2ΔLRR (Supplementary Fig. 2) was produced by applying the instructions kit of *Gel Filtrations Markers Kit for Protein Molecular Weights 29,000-7000,000* *Da*, (Sigma). To determine the Void Volume elution (VV), blue dextran was dissolved at concentration of 2 mg ml^−1^ in 20 mM Tris pH 8, 50 mM NaCl, 1 mM TCEP and 5% Glycerol. Proteins Standards were mixed at ratio indicated in the instructions, in the same blue dextran buffer. 300 ul of each sample were applied to the column, at 0.5 ml min^−1^. The gel filtration runs were performed at room temperature in buffer containing 20 mM Tris pH 8, 50 mM NaCl and 1 mM TCEP.

### In vitro radioactive phosphorylation assay

We used an in vitro radioactive assay to analyse the auto-phosphorylation activity of recombinant RIP2fl^36^. 1.2 µg of freshly purified RIP2fl were mixed with 10 µM ATP (10:1 ATP-gamma-^32^P) and 10 mM MgCl_2_. The reaction was incubated at 30 °C and blocked at 1, 3, 5, 10, 15 min by adding SDS loading buffer. Resulting samples were applied to 12% SDS-PAGE gel and results were revealed using a Typhoon scanner (GE Health).

### Liquid chromatography/electrospray ionization mass spectrometry

The phosphorylation profile of freshly purified and non-aggregated RIP2fl by Liquid Chromatography/Electrospray Ionization Mass Spectrometry (LC/ESI-MS), was analysed on a 6210 TOF mass spectrometer coupled to a HPLC system (1100 series, Agilent Technologies)^36^. The mass spectrometer was calibrated with tuning mix (ESI-L, Agilent Technologies) and the following settings were used: gas temperature (nitrogen) 300 °C, drying gas (nitrogen) 7 L min^−1^, nebulizer gas (nitrogen) 10 psig, Vcap 4 kV, fragmentor 250 V, skimmer 60 V, Vpp (octopole RF) 250 V. The HPLC mobile phases were prepared with HPLC grade solvents. Mobile phase A composition was: H_2_O 95%, ACN 5%, TFA 0.03% whilst mobile phase B was ACN 95%, H_2_O 5%, TFA 0.03%.

Two samples were analyzed: freshly purified and non-aggregated RIP2fl (phosphorylated RIP2fl) and the same RIP2fl sample supplemented with 0.3 U of lambda protein phosphatase (New England Biolabs) per μg of protein (de-phosphorylated RIP2fl). The second sample was measured after 1.5 h of incubation at room temperature. Using a C8 reverse phase micro-column (Zorbax 300SB-C8, 5μm, 5 × 0.3 mm, Agilent Technologies) the protein samples were desalted on-line for 3 min at 100 μl min^−1^ with 100% of mobile phase A. After elution at 50 μl min^−1^ with 70% of mobile phase B, spectra were acquired in the positive ion mode in the 300-3000 *m/z* range. Data were processed with MassHunter software (v. B.02.00, Agilent Technologies) and the number of RIP2fl phosphorylation sites was calculated by comparing the two spectra obtained.

### Negative-stain EM

Two different protocols were used to prepare negative-stain grids. For RIP2fl sample, 4 µl of sample were applied to the clean side of the carbon on a carbon-mica interface, letting the sample absorb for 20 s. The carbon film was then floated on a drop of 2% (w/v) uranyl acetate, picked up with a 400-mesh copper grid (Electron Microscopy Science) and dried on filter paper (Whatman).

For all the other samples, 6 µl of protein solution were applied to glow-discharged carbon coated copper grid (300 mesh, Electron Microscopy Science) and let adsorb for 30 s. Grids were then washed twice in 25 µl drop of protein buffer and stained twice for 30 s with 6 µl of 2% (w/v) uranyl acetate. Between each step excess of protein/buffer solution/staining was blotted off using a filter paper. Grids were dried on adsorbing paper for at least 5 min before storage^55,56^.

Negative-stain grids were imaged either with a JEOL 1200-EX II microscope at 100 KV on photographic film or with a Tecnai 12 (FEI) TEM at 120 KV on a Ceta 16 M camera, at a nominal magnification of 16,000× to 48,000×.

Negative-stain grids prepared to investigate the effect of nucleotides on RIP2 polymerization (see below), were imaged using a Tecnai 12 (FEI) at a nominal magnification of 30,000×.

### Polymerization of MBP-RIP2fl and RIP2fl

Polymerization of RIP2fl was induced by mixing 20 μl of purified tag-free RIP2fl at 0.3 mg ml^−1^ with 5 mM ATP (Sigma) and 10 mM MgCl_2_. After one hour of incubation at room temperature, sample was visualised by negative-stain EM.

In order to assay the relevance of ATP for RIP2 polymerization, MBP-RIP2fl was purified as RIP2fl, omitting the tag cleavage step. Protein was concentrated until 1 mg ml^−1^ in final buffer 20 mM Tris pH 7.5, 50 mM NaCl and 2 mM βMe. Nucleotides (ATP, AMPPCP ADP, AMP, from Sigma) were prepared as follows: nucleotide stocks were dissolved in Milli-Q water at 100 mM and stored at −80 °C. For each experiment a new aliquot was quickly thawed and diluted twice in 1 M Tris pH 7.5, 100 mM MgCl_2_ and successively diluted 10 times in protein sample. MBP-RIP2fl was then incubated with 5 mM ATP, ADP, AMP or AMPPCP (Beta,gamma-methylene-adenosine 5’-triphosphate) overnight at room temperature. As controls, we also prepared MBP-RIP2fl and MBP-RIP2fl supplemented with 10 mM MgCl_2_. For each sample one negative-stain grid was prepared and 10 micrographs collected. The entire experiment was repeated twice.

### Structure determination of crystMBP-RIP2CARD

Freshly purified crystMBP-RIP2CARD was concentrated to 6 mg ml^−1^ and used immediately for crystallization trials. Initial crystallization conditions were established by testing several commercial screens at the EMBL High Throughput Crystallization Laboratory (Grenoble, France) using a Cartesian robot. The best crystals were obtained at 4 °C with the sitting drop method from solutions containing 6 mg ml^−1^ of crystMBP-RIP2CARD equilibrated against 0.25 M NaNO_3_, and 22% (w/v) PEG 3350. Crystals were transferred to a cryo-protection buffer prepared with mother liquor supplemented with 20% (v/v) glycerol, plunged into liquid nitrogen and stored. Diffraction data were collected on ID29^57^ at the ESRF (Grenoble, France) at a wavelength of 0.976 Å and temperature of 100 K. Data were processed and scaled with XDS^58,59^. The structure was solved by molecular replacement using PHASER^60^ with the NLRP1 CARD domain with a crystallisable MBP at the N-terminus (PDB accession code 4IFP) as search model. There are four fusion proteins per asymmetric unit. Refinement was carried out using cycles of REFMAC5^61^ and manual rebuilding with COOT^62^. Local NCS constraints and TLS refinement (one set for each MBP and each CARD) were used. Models were validated using Molprobity^63^ from the PHENIX interface. Final statistics are: Ramachandran: 95.57% allowed, 0.44% outliers; 2.3% rotamer outliers; Clashscore 1.62; Molprobity score 1.49. Ramachandran outliers are in the MBP molecule structure. Structure figures were produced with PyMol^64^. Data collection and refinement statistics are in Supplementary Table 1. RMSD calculations were run with Superpose^65^, using the option: Secondary structure matching.

### Sample preparation for solid state NMR

RIP2CARD (435–540) cloned into pETM40 (KanaR) was transformed into BL21 star (Thermo Fisher Scientific) for expression. All LB precultures contained 2% glucose to repress the lac operon. NMR-specific labels (all isotopes from Eurisotop) were introduced by expression at 22 °C overnight in 1.5 fold M9 medium following the 2-fold concentration method^66^. For uniform ^13^C and ^15^N labelling, M9 medium was supplemented with 0.7 g L^−1 15^N NH_4_Cl and 2.5 g L^−1 13^C-glucose. For sparse labelling with [2-^13^C]- or [1,3-^13^C]-glycerol, to measure long range constraints, the protocol previously described^67^ was used. Deuteration was achieved by using D_2_O instead of H_2_O for all medium components.

Purification was done in the presence of 2 M urea to prevent aggregation or too early filament formation as reported^40^. Cells from 1.2 L culture resuspended in 20 mM Tris pH 8, 20 mM NaCl, 2 M urea, 2 mM β-Me, 1 mM Pefabloc (Sigma), 5 mM MgCl_2_, and 15 µL Benzonase (Merck) were disintegrated by high pressure (Microfluidizer LM10, Microfluidics). After centrifugation at 22,000 × *g*, for 1 h at 4 °C, the supernatant was filtered (0.45 µm) and incubated with about 12 ml 50 % amylose resin (New England Biolabs) at 15 °C on a rotator for 3 h. MBP-RIP2CARD was eluted with 10 mM maltose in 20 mM Tris pH 8, 20 mM NaCl, 2 M urea, 2 mM β-Me after a washing step. The fusion protein, at about 1 mg ml^−1^, was cleaved by TEV protease overnight at 22 °C. The next night was used for dialysis against 20 mM Tris pH 8, 20 mM NaCl, 2 mM β-Me at 8 °C to remove urea and to begin filament formation lasting a further 48 h at 20 °C. Filaments were collected by ultracentrifugation at 35000 × g for 1 h to produce a pellet for solid state NMR and quality checking by negative-stain EM.

### Solid state NMR

The RIP2CARD filaments were packed into the respective rotor by ultracentrifugation at 100000 × g for 1 h using a custom-made filling device.

All proton-detected experiments were recorded on a wide-bore 800 MHz spectrometer equipped with a 1.3 mm triple-resonance MAS probe (Bruker, Karlsruhe, Germany). Typical π/2-pulse lengths were 2.5 μs for ^1^H, 5 μs for ^13^C and 7 μs for ^15^N. The MAS frequency was set to 60 kHz and the sample temperature was kept at approximately 295 K. For the backbone assignment, a standard set of experiments, (H)CANH, (HCO)CA(CO)NH, (HCA)CB(CA)NH, (HCA)CB(CACO)NH, (H)CONH and (H)CO(CA)NH, was recorded on ^2^H, ^13^C, ^15^N-labeled and 100% back exchanged RIP2CARD filaments. Water suppression was achieved using the MISSISSIPI sequence and WALTZ-16 was used for ^13^C and ^15^N decoupling during proton detection. The spectra were processed with NMRPipe employing shifted-sinebell and Lorentzian-to-Gaussian apodization functions.

The ^13^C-^13^C DARR correlation spectra were recorded on wide bore 600 and 700 MHz spectrometers equipped with 3.2 mm triple-resonance MAS probes (Bruker, Karlsruhe, Germany). Typical π/2-pulse lengths were 3.1 μs for ^1^H, and 5 μs for ^13^C. All 2D spectra were recorded at either 13 333 (on the 600 MHz) or 15 555 Hz (700 MHz) MAS frequency and a sample temperature of approximately 285 K. Various mixing times, with durations of 10, 50, 150, 300 and 500 ms were used for the 2-glycerol and 1,3-glycerol-labelled RIP2CARD samples, whereas DARR mixing times of 10 and 50 ms were used for the uniformly ^13^C-labelled sample. During acquisition and indirect chemical shift evolution a SPINAL64 decoupling scheme with a RF strength of 90 kHz was applied to the proton spins. The data were processed using Topspin version 3.2 (Bruker, Karlsruhe, Germany) applying shifted-sinebell and Lorentzian-to-Gaussian apodization functions. All spectra were recorded using Topspin and were further analyzed with the CCPN Analysis v.2.4.2 package^68^.

### Immuno-gold labelling

For immuno-gold labelling experiments, we modified protocol illustrated in *Lu, A et al., 2015*, as follows. To detect the interaction between NOD2ΔLRR and RIP2CARD filaments, purified NOD2ΔLRR was mixed with a 50-fold excess of MBP-RIP2CARD and protein polymerization was induced by adding TEV to cleave off the tag. To detect the interaction between NOD2CARDS and RIP2CARD filament, HIS-SUMO-NOD2CARDS^s^ and MBP-RIP2CARD were co-purified as described above. Immuno-gold labelling was applied after MBP and HIS-SUMO tags cleavage without further purification.

For immuno-gold labelling, 6 µl of sample were applied to glow-discharged carbon coated copper grid (300 mesh, Electron Microscopy Science) and let adsorb for 30 s. Excess sample was blot off on filter paper, and grid was let shaking 10 min in 30 µl drop of PBS + 0.1% (w/v) BSA. After removal of excess, grid was transferred to 30 µl drops of same solution containing either the rabbit antibody anti-SNAP at dilution 1:2000 (P9310, New England Biolabs), or rabbit anti-NOD2 at dilution 1:1000 (ab197030, Abcam) or no antibody (control sample C2). The specimen was then left in 30 µl PBS + 0.1% (w/v) BSA for 1 min and the same washing procedure was repeated three times. Successively, the grid was floated for 1 h in a 30 µl drop of the same solution containing gold-conjugated goat antibody anti-rabbit at 1:100 dilution (G7277, Sigma). After washing a second time, the grid was stained with 2% (w/v) uranyl acetate and images were recorded on a JEOL 1200 at 16,000x or 25,000x magnification.

### Grid preparation for cryo-EM and cryo-EM data collection

The sample quality and filament concentration were checked by negative-stain EM prior to cryo-EM grid preparation. Grids for cryo-EM were coated with a thin layer of carbon as follows. A piece of homemade carbon-mica foil was floated for 5 min on a 30 µl sample on an ice-cooled home-made polyoxymethlene block. A Quantifoil R2.2 grid was glow-discharged for one minute at 25 mA (PELCO easy glow), applied on top of the floating carbon foil and quickly transferred to a Vitrobot Mark IV (FEI) for vitrification. Vitrobot filter papers (TED PELLA, INC.) were humidified prior to use and the Vitrobot was set to 4 °C and 100% humidity. A drop of 3 µl water was added to the specimen prior to blotting at a force of -5 for a total time of 45 s. The grid was finally flash-frozen in liquid ethane before storage.

Cryo-EM movies where automatically collected on an FEI Polara electron microscope operated at 300 KV. Micrographs were recorded on a K2 direct electron detector operated in counting mode using the software Latitude S, at a nominal magnification of 41270 × (corresponding to 1.21 Å/pixel at the specimen level), with a defocus range of -1.5 to -3μm. 720 movies of 40 frames were collected with a total dose of 50 electrons Å^−2^ (Supplementary Table 3).

### Image processing, symmetry determination and structure refinement

After selection according to ice quality, half of the movies collected were actually used for data processing. The frames 2 to 16, corresponding to a total dose of 20 electrons Å^−2^, were motion-corrected and dose-weighted using MotionCor2^69^. The defocus estimation was performed with CTFFIND/CTFTILT^70^.

Initially, 4125 sections of filaments (total length 97 µm) were manually boxed using the e2helixboxer module of EMAN2^71^, while avoiding to include the filament-ends where the hetero-CARD complex could be situated. This manually picked data set was analysed with RELION^72^, to obtain 2D class-averages used both as templates for automatic picking, and for initial symmetry estimation from their power spectrum. To this end, the filaments were segmented with an inter-box distance of 25 Å into 23742 segments of size 420*420 pixels, which were used for 2D classification, asking for thirty classes. Three significantly populated 2D class-averages were obtained, amongst which two correspond to straight segments, and show a clear repetitive pattern along the helical axis (Fig. 6b). The individual power spectra of these two 2D class-averages were nearly identical, and showed layer lines up to ~1/4.9 Å^−1^ (Fig. 6c), which enabled a first estimation of the helical parameters as follows (Supplementary Fig. 8a). The layer lines are spaced regularly at multiples of ~1/155.8 Å^−1^, giving an estimation of the repeat c. A meridional layer line l = 32 (Bessel order n = 0), with a height of 1/4.87 Å^−1^ indicated the axial rise between subunits. A strong layer line l = 9 with a first intensity maximum near the meridian (n = 1), at 1/17.3 Å^−1^, was attributed to the pitch P, as observed for other CARD domains filamentous assemblies (Supplementary Table 4). Therefore, the structure repeats after u = 32 subunits (32 * 4.87 = 155.8), in t = 9 turns, resulting in a number of units per turn (u/t) of ~3.56.

The two straight 2D class-averages were subsequently used as templates for automatic picking of helical segments in RELION, using a maximum curvature parameter κ = 0.4, and a minimum filament length of 800 Å. The coordinates of filament extremities were converted into EMAN2 format, while shortening them by 180 Å at each extremity, to avoid including the hetero-CARD complex. This resulted in 4443 filament sections for a total length of 260 µm.

All subsequent processing steps were performed in the helical reconstruction software package SPRING^73^. An initial model was obtained by 3D refinement from the manually picked data set and the symmetry parameters as estimated from the 2D class average power spectrum indexing, with low-pass filtering to 20 Å. The auto-picked filament coordinates were used to extract 37043 segments using a segment length of 400 Å and segment step size of 70 Å. The symmetry parameters were further refined with the module Segrefine3Dgrid, by defining a 11*11 grid spanning between 17.2-17.4 Å (step 0.02 Å) for the pitch and between 3.4–3.6 (step 0.02) for the number of units per turn. The maximum of the amplitude correlation between experimental and reprojection power spectra was found at a pitch of 17.26 Å, 3.56 units per turn (Supplementary Fig. 8b), corresponding to a left-handed helix with -101.124° helical twist and 4.848 Å axial rise. Using the refined symmetry parameters, we performed a high-resolution structure refinement using the auto-picked segments with a strict segment selection during refinement, based on geometrical restraints^73,74^ namely filament straightness (70% of straightest filaments kept) and forward x-shift difference (limited to 5 Å). This resulted in a final reconstruction including 9661 segments (corresponding to 135,254 asymmetric units after symmetrisation) at a resolution of 3.94 Å (FSC between half data set maps, cut off 0.143, Supplementary Fig. 8c). For visual display and the model building, the EM map was filtered to 3.9 Å and sharpened using a B-factor of −200 Å^2^.

Visualization of the resulting map and initial rigid body fitting of the crystallographic structure was done using Chimera^75^. Atomic model refinement was done using PHENIX real space refinement^11^ and manual adjustment with Coot^62^. The crystallographic model was used to assign side chains for the residues without clear EM density map. Structure validation was done with Molprobity^63^. Figures were prepared with PyMOL^64^ and Chimera. Software used in this project was installed and configured by SBGrid^76^. Data collection, image processing and refinement statistics are reported in Supplementary Table 3 and Supplementary Fig. 8d.

### Production of RIP2CARD mutants and Western blots

RIP2CARD mutants were produced by PCR mutagenesis of the HIS-MBP-RIP2CARD construct, using the oligos and their complements as reported in Supplementary Table 5. Expression was performed as described above.

For co-production of RIP2CARD mutants with NOD2CARDS^s^, 0.250 ml (~ 2.5 g of bacterial pellet) of HIS-MBP- RIP2CARD mutants culture was combined with 100 ml of NOD2CARDS^s^ (~ 9 g of bacterial pellet) and resuspended in 50 ml of lysis buffer (20 mM Tris pH 8, 50 mM NaCl, 1 mM TCEP and Complete protease inhibitor cocktail (Roche)). The sample was then treated as described for NOD2CARDS^s^-RIP2CARD filament sample. After elution from the amylose resin, complex formation was checked on SDS-PAGE, and presence of HIS-SUMO-NOD2CARDS^s^ confirmed by western blot (WB). For WB, 8 µg of total protein was loaded on either a 17% SDS-PAGE gel (Fig. 5b) or stain-free 4–12 % gradient SDS-PAGE gel (Bio-Rad) (Fig. 8b). Rabbit anti-SNAP at 1:2000 dilution (P9310,New England Biolabs) was used for detection of NOD2CARDS^s^. For WB revelation, a goat anti-rabbit secondary antibody linked to Alkaline Phosphatase was used at 1:30000 dilution (A3687, Sigma). Uncropped blots are shown in Supplementary Fig. 13.

Filament polymerization was stimulated by adding proteases for 4 h at room temperature, at a protein concentration of 2.5 mg ml^−1^. Negative-stain EM was used to check the polymerization state of each sample.

Experiments were repeated twice for constructs that showed impaired RIP2CARD polymerization.

### Mammalian cell culture and plasmids

A HEK293T cell line (from the laboratory of W.Filipowicz) was used. The cell line has not been authenticated but has been tested and shown to be free of mycoplasma. Cells were maintained in DMEM medium (Lonza) supplemented with 10% (v/v) fetal bovine serum (FBS) and non-essential amino acids (Gibco), at 37 °C and 5% CO_2_. Constructs for in cell based assays, were generated from pcDNA3 plasmids encoding for human RIP2fl^50^. The original pcDNA3-RIP2(10–540) was modified into pcDNA-HA-RIP2(1–540), using the oligos reported in Supplementary Table 5, which resulted in a construct encoding for full-length RIP2 with an HA tag at the N-terminus and a GSAGSA linker between tag and protein. Single amino acids mutants were obtained by site-directed PCR mutagenesis of pcDNA3-HA-RIP2(1–540), using the oligos listed in Supplementary Table 5.

### In cell reported luciferase assay and Western blots

HEK293T cells were seeded in 12-well plates 24 h prior transfection. Transfection was performed with LipoD293 transfection reagent (SignaGen). Each well was transfected with 5 ng pcDNA3 plasmid encoding for pcDNA3-HA-RIP2 or corresponding mutant, 1 ng of pCDNA3, 500 ng of Luc-NF-κB^50^, encoding a firefly luciferase reporter gene under NF-κB promoter and 50 ng of pRenilla-TK plasmid. As negative control, transfection mix with pcDNA3-HA-RIP2 replaced with empty vector was used. pRenilla-TK was used to correct for the transfection efficiencies. Each transfection mixture was prepared in double and completed with either the NOD2 activator MDP or its corresponding control cMDP (Invivogen). Cells were lysed 20 h after transfection in 250 µl of lysis buffer, accordingly to the manufacturer protocol Dual Luciferase assay (Promega). Sample was then used for luciferases activities measurement and for western blots (WB). Firefly and Renilla luciferase activities were measured using a Clariostar microplate reader using the double injector system (BMG Germany). 10 µl per sample were dispensed in a luciferase specific 96-wells plate (Thermo Scientific). 25 µl of Firefly developing solution (LucII) were firstly injected, the plate was stirred at 500 rpm for 15 s, and 12 measurements performed. 25 µl of Stop and Glow solution were then dispensed. The plate was stirred again and the same number of measurements were recorded. Each experiment was repeated three times (biological replicate) and each time, three transfections per condition were performed (technical replicate), for a total of nine experiments per construct.

For WB, 10 µg of total protein were loaded on a stain-free 4–12 % gradient SDS-PAGE gel (Bio-Rad). Mouse anti-HA (26183, Thermo Fisher) at 1:2000 dilution was used for detection of transfected HA-RIP2 and its corresponding mutants, whilst anti β-actin antibody at 1:2000 dilution was employed for normalization of total protein amount (ab8227, Abcam). For WB revelation secondary antibodies linked to fluorophores were used 1:2000 dilution, goat anti-mouse and goat anti-rabbit linked to Alexa 532 and Alexa 633 respectively (A11002 and A21070 respectively, Thermo Fisher). A representative WB was run for each biological replicate. Uncropped blots are shown in Supplementary Fig. 14 and 15.

### Statistical analysis

Statistical analysis on the luciferase reporter assay was performed using Rstudio^77,78^ with the aim of quantitatively finding evidence of significant differences between the two experiments, namely with NOD2 ligand MDP and the control cMDP. Their mean response was assessed using a parametric method; hence each of the samples was evaluated using a Shapiro Wilk test for normality. The null hypothesis assumed normality, and all samples failed to reject this hypothesis test showing at 95% of significance that they were all normally distributed. Moreover, samples were assumed to have homogenous variances to satisfy the requirements of the parametric tests. The Student *t-*test on two independent samples (two-sided) was conducted for each pair of conditions. Firstly, wild-type RIP2 and vector showed, without ambiguity according to p-value, that their respective means were different. Then, wild-type RIP2 was compared to the mean of each mutant. Except for wild-type RIP2 vs mutant Q458A, Q458K, N512A and N512K where no significant differences from their means was observed, all other mutants rejected more or less strongly the null hypothesis. In other words, their mean responses were significantly different from that of wild-type RIP2. Finally, the Student *t*-test comparison of two independent samples were conducted on “MDP” and “cMDP” version of each mutants, including both vector and wild-type RIP2. All conditions rejected the null hypothesis; leading to the conclusion that their means were different at 95% significance. Once quantitative evidence about differences from MDP and cMDP experiment was confirmed, a boxplot was generated to visualise the results (Fig. 9).

## Electronic supplementary material

Supplementary Information

## Data Availability

Coordinates and structure factors for crystMBP-RIP2CARD are deposited in the PDB with accession code 6GFJ [https://www.rcsb.org/structure/6GFJ]. Coordinates for the RIP2CARD filament have accession code 6GGS [https://www.rcsb.org/structure/6GGS]. The cryoEM map has accession code EMD-4399. The solid-state NMR data is deposited in the BMRB database with accession number 27555 (this data can be retrieved using the restart ID 2018-07-16.deposit.bmrb.wisc.edu.80.13422729 at http://deposit.bmrb.wisc.edu/bmrb-adit/access.html).
