# Supplementary material for: RIP2 filament formation is required for NOD2 dependent NF-κB signalling
Source: Nat Commun. 2018 Oct 2;9:4043. doi: 10.1038/s41467-018-06451-3 (PMC6168553; doi:10.1038/s41467-018-06451-3)
Supplement: Supplementary file 1 — Supplementary Information [file 41467_2018_6451_MOESM1_ESM.pdf]

Supplementary Information

Pellegrini et al. **RIP2 filament formation is required for NOD2 dependent NF-κB signalling**

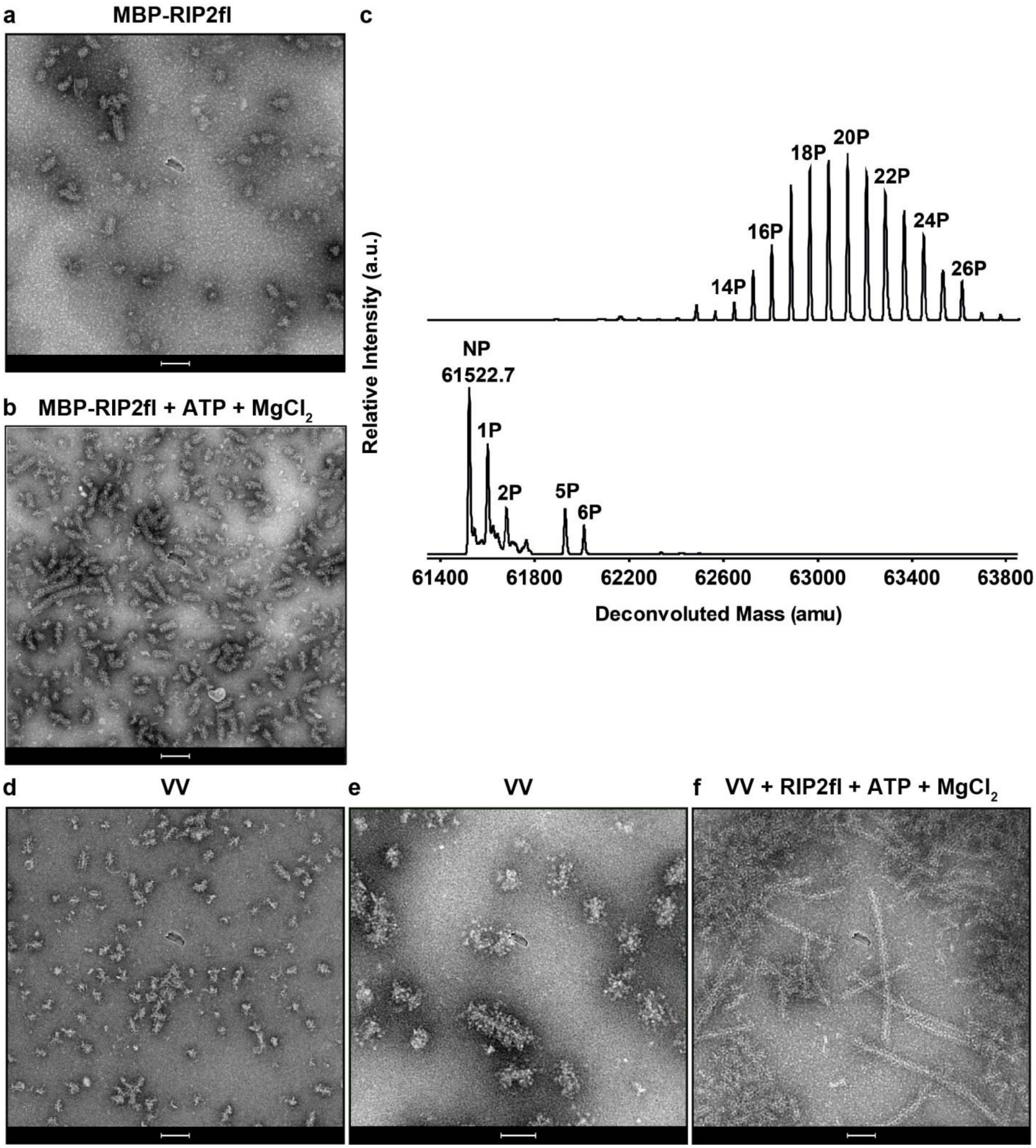

Supplementary Figure 1: Phosphorylation profile of recombinant RIP2fl by ESI-TOF mass spectrometry

a-b: Negative-stain micrographs of (a) MBP-RIP2fl from the amylose resin eluate, (b) MBP-RIP2fl from the amylose resin eluate plus ATP and magnesium. Scale bars are 100 nm.

c: The mass spectra of recombinant tag-free RIP2fl was acquired by electrospray ionization time-of-flight mass spectrometry (ESI-TOF MS). Deconvoluted mass spectra are shown for RIP2fl immediately after purification (top) and after lambda phosphatase treatment (bottom).

d-f: Negative-stain micrographs of (d) RIP2fl from the column void volume (VV), (e) zoomed-in view of RIP2fl from the VV, (f) VV RIP2fl plus 5-fold of non-aggregated RIP2fl, ATP and magnesium. Scale bars are 100 nm, 50 nm and 100 nm respectively.

a

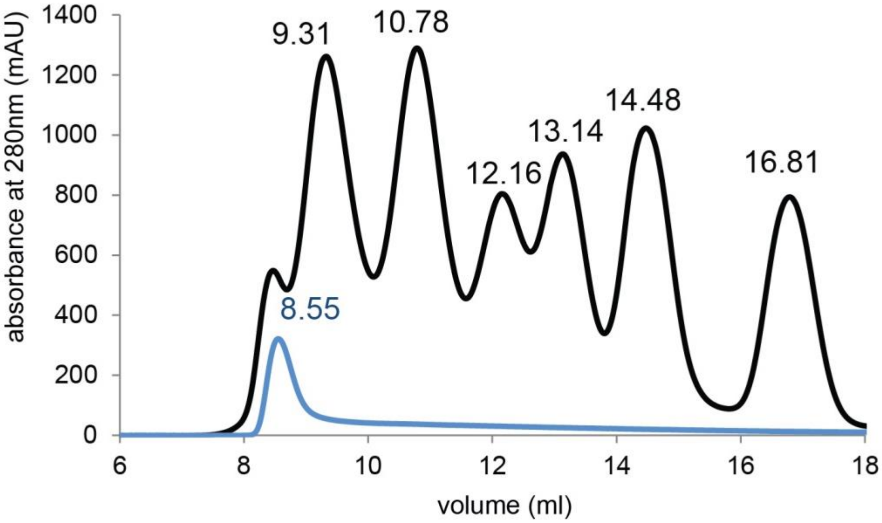

b

Proteins	VV		
Blue Dextran	8.55 ml		
	Ve (ml)	Ve/VV (ml)	MW (KDa)
Thyroglobulin	9.31	1.09	669
Apoferritin	10.78	1.26	443
Amylase	12.16	1.42	200
Alcohol deydrogenase	13.14	1.54	150
Albumin	14.48	1.69	66
Carbonic Anhydrase	16.81	1.97	29

Supplementary Figure 2: Calibration curve of Superdex 200 gel filtration column

a: Calibration curve of Superdex 200 gel filtration column, showing the elution volume (Ve) of blue dextran (in blue) and protein standards (in black).

b: Table showing the correspondence between each standard protein, elution volume (Ve), Ve/VV ratio and molecular weight (MW) expected.

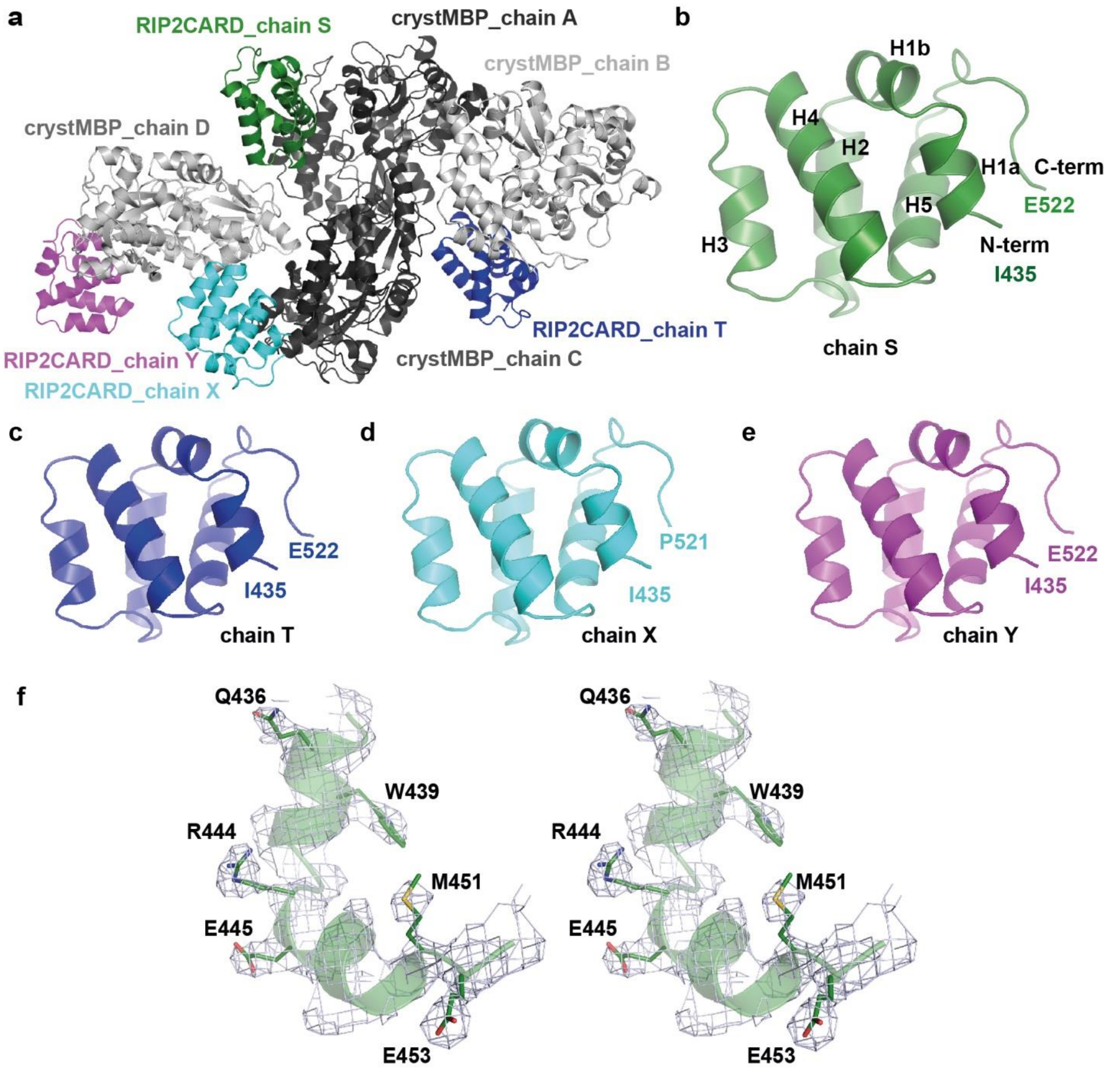

Supplementary Figure 3: Overview of crystMBP-RIP2CARD asymmetric unit

a: Ribbon diagram of the contents of the crystMBP-RIP2CARD asymmetric unit, which comprises four MBP subunits (chains A, B, C, D), each fused to a RIP2CARD molecule (chains S, T, X ,Y).

b-e: Ribbon diagrams of the different RIP2CARD chains in the asymmetric unit: (b) chain S, (c) chain T, (d) chain X and (e) chain Y. All the chains ends with E522, except chain X which ends at P521.

f: Stereo image of the 2Fo-Fc map, contoured at 1.5 σ, showing the helices 1 and 2 of RIP2CARD-chain S (residues 435-454).

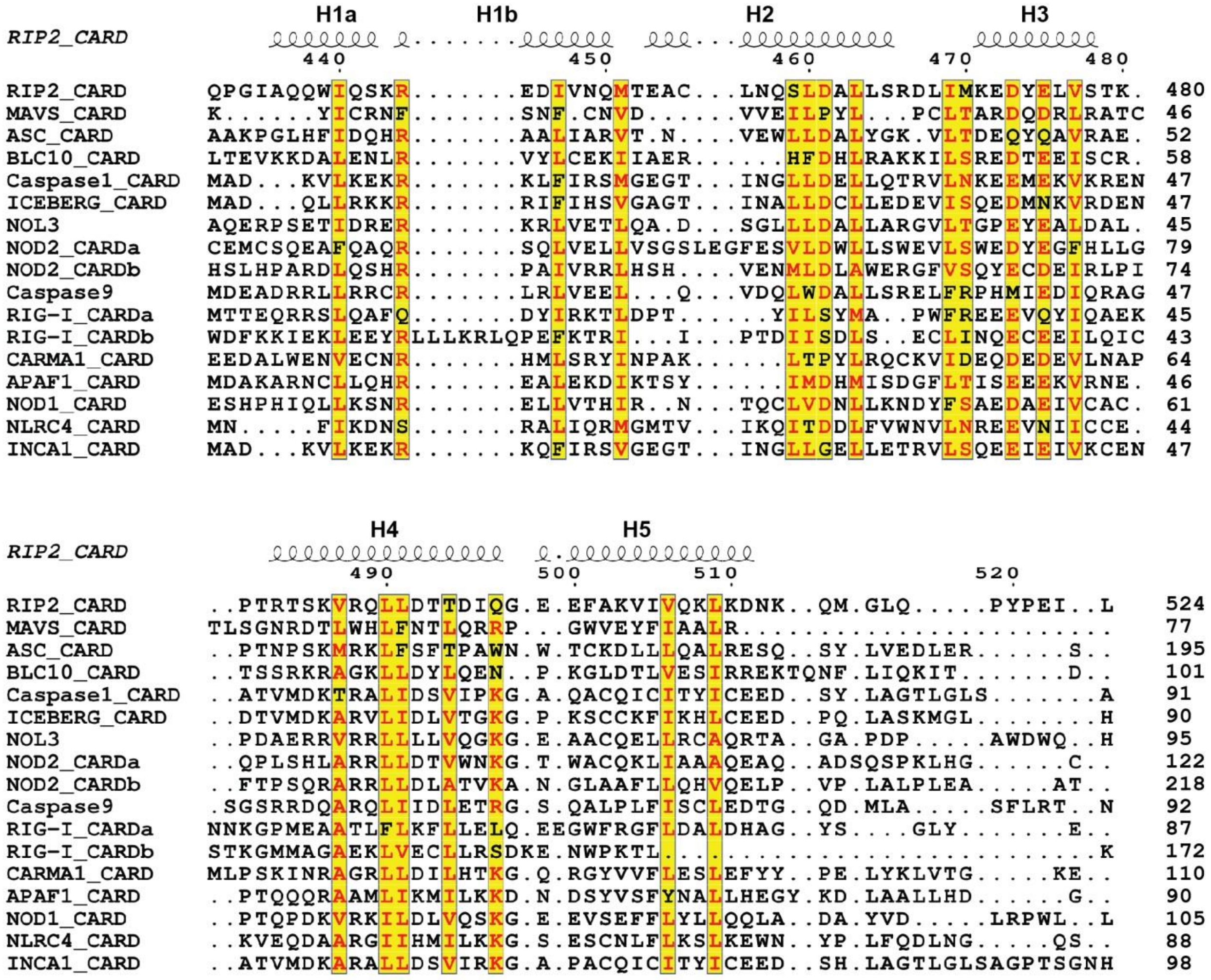

Supplementary Figure 4: Structure-based sequence alignment of relevant human CARDs.

Structure-based alignment of CARDs belonging to receptors and adaptor proteins of the innate immune system. The alignment was computed using the structure-based multiple-sequence alignment programme 3DCoffee[1] for which PDB models are automatically selected. EXPRESSO output was used as input for ESPript 3.0[2], to generate the figure. The secondary structure assignment of the RIP2CARD crystal structure is shown on top. Similar residues are red and highlighted in yellow.

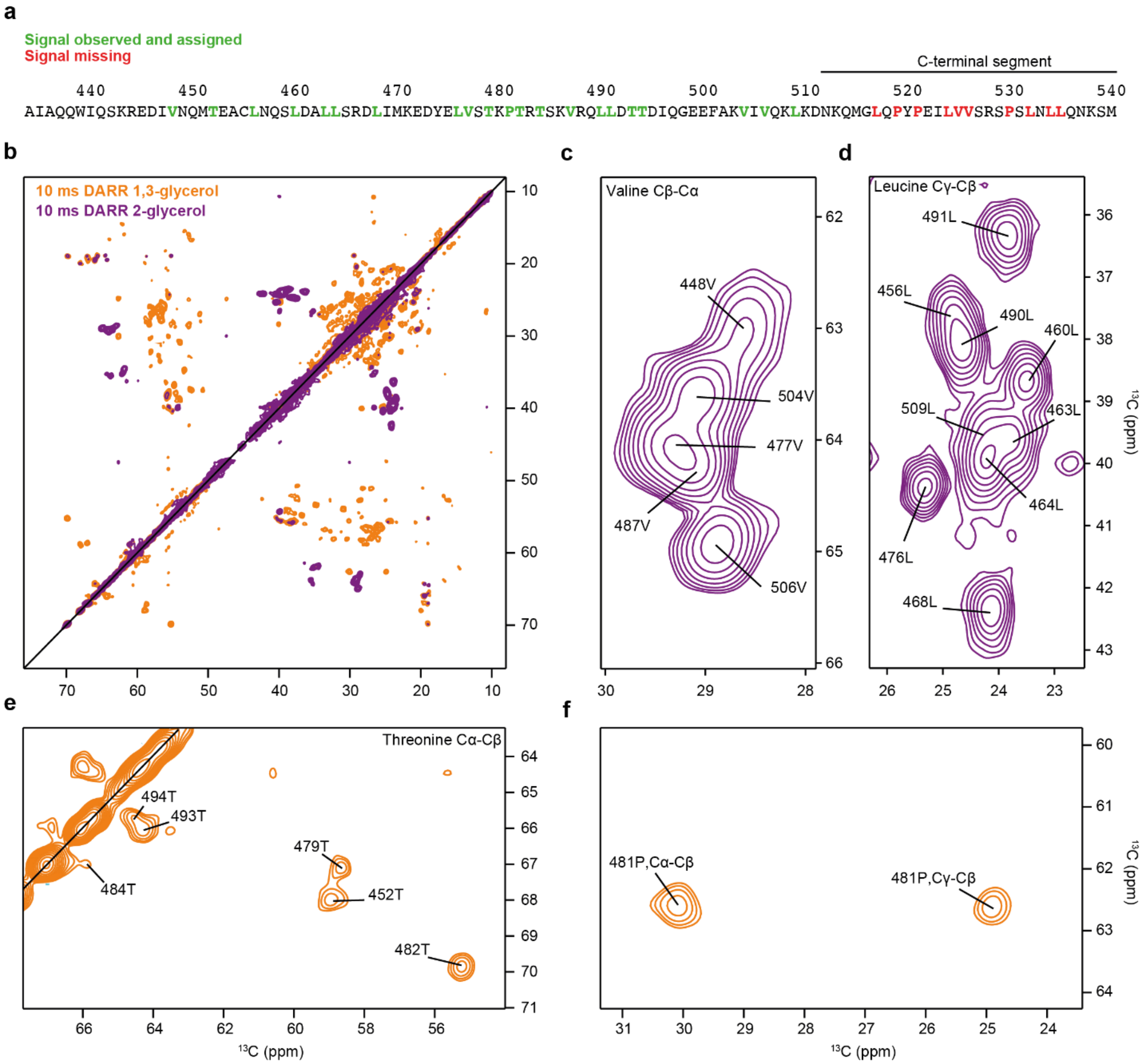

Supplementary Figure 5: The signals of the RIP2CARD C-terminus are missing in ^{13}C-detected solid-state NMR experiments.

a: Sequence of RIP2CARD with residues highlighted, that show assigned cross-peaks in the ^{13}C-detected NMR spectra (green) and residues that are missing in the spectra (red) corresponding to the C-terminal segment of the protein (512-540).

b: Superposition of 10 ms DARR spectra of 1,3-glycerol-labelled (orange) and 2-glycerol-labelled (purple) RIP2CARD.

c-f: Sections of the spectra in (b), highlighting the regions that include the characteristic cross peak patterns of (c) valine, (d) leucine, (e) threonine and (f) proline residues. Sequence-specific assignments are indicated.

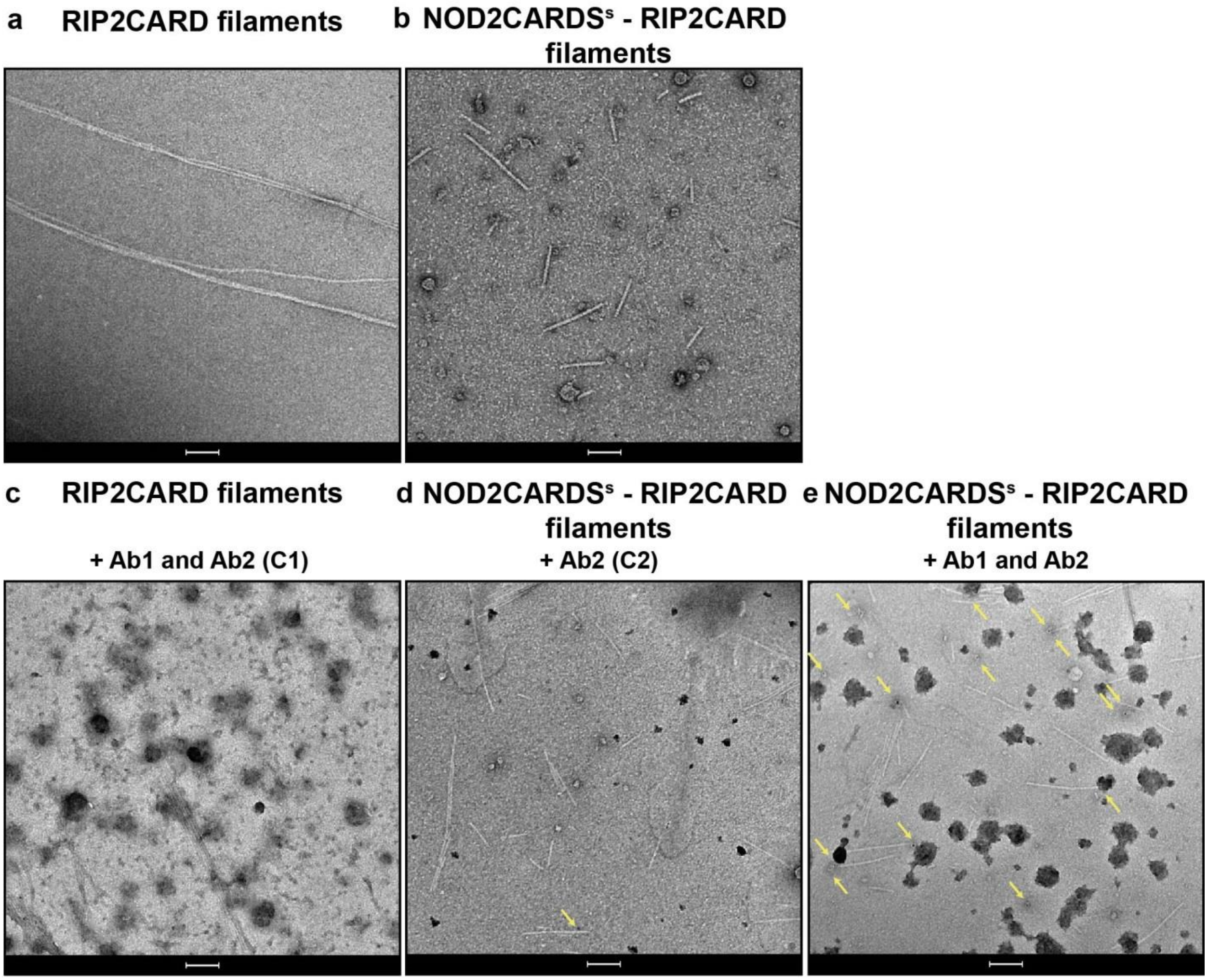

f

Sample	Rip2CARD filaments	NOD2CARDS^s + Rip2CARD filaments	NOD2CARDS^s + Rip2CARD filaments
ANTI-SNAP	+	-	+
gold-ANTI-RABBIT	+	+	+
total number of gold particles	81	15	227
number of gold-particles on filaments	9.9 %	13.3%	70.9%
number of gold-particles on filament-ends	gold-particles to aggregated filaments	only one gold particle bound to filaments	91.7% (of number of gold particles on filaments)

Supplementary Figure 6: Analysis of the binding position of NOD2CARDS^S on RIP2CARD filaments.

a-b: Negative-stain images of (a) RIP2CARD filaments and (b) NOD2CARDS^S -RIP2CARD filaments. Samples (a) and (b) were used for immuno-gold labelling experiments (see Figure 5).

c-e: Representative micrographs showing gold-particle counting for (c) RIP2CARD filaments sample (Control 1, C1), (d) NOD2CARDS^S -RIP2CARD filaments without first antibody (Control 2, C2), (e) NOD2CARDS^S -RIP2CARD filaments. Gold-particles are indicated by yellow arrows. (Ab1=anti-SNAP, Ab2=gold-anti-rabbit).

f: Statistics on gold-particle counting. Total number of particles and percentage bound to filaments/filament-ends are reported for each condition analysed.

Scale bars are 100 nm.

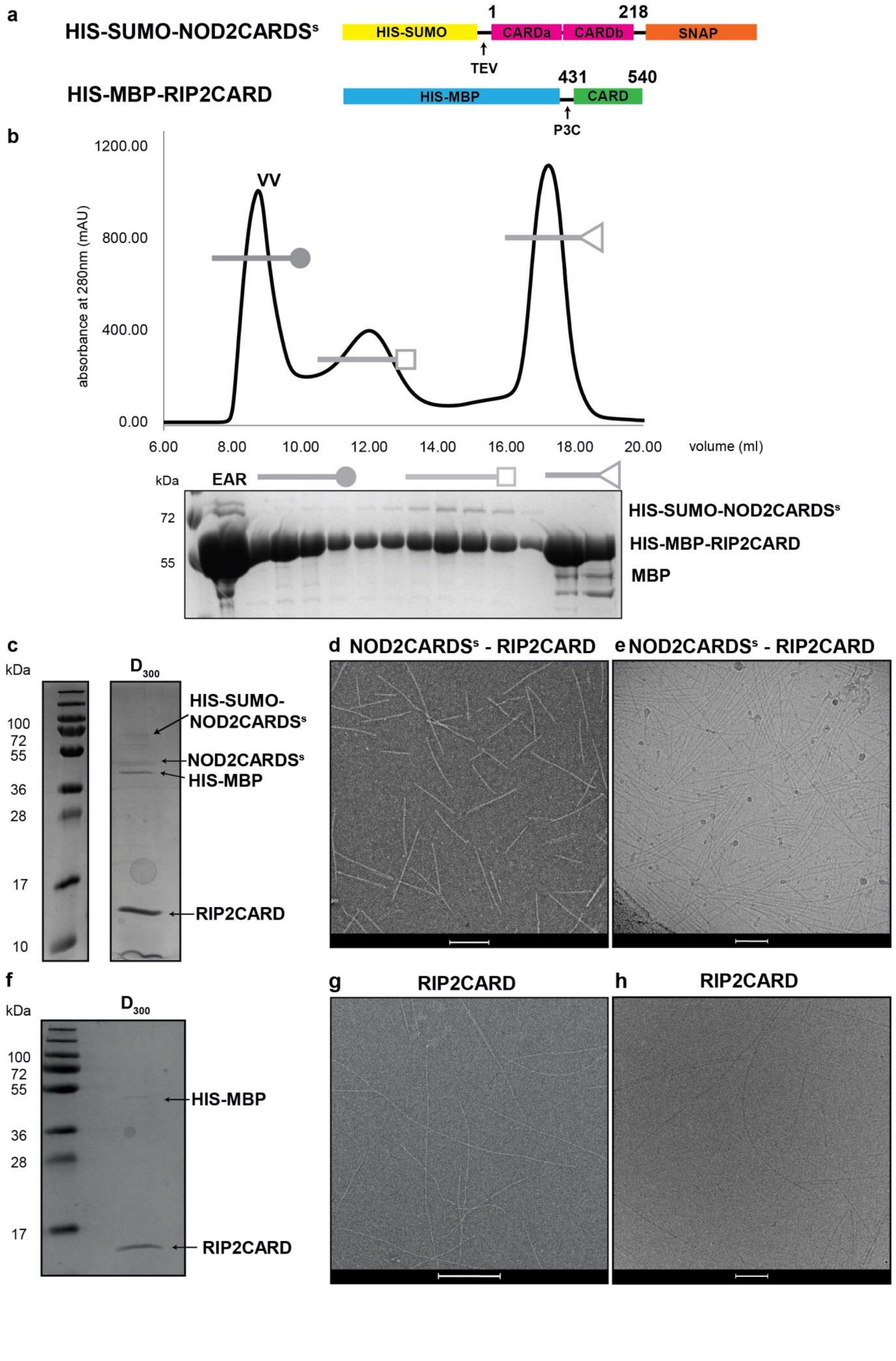

a
HIS-SUMO-NOD2CARDS^{s}
HIS-SUMO
CARDa
CARDb
SNAP
1
218
TEV
HIS-MBP-RIP2CARD
HIS-MBP
CARD
431
540
P3C
b
VV
absorbance at 280nm (mAU)
volume (ml)
kDa
EAR
72
55
HIS-SUMO-NOD2CARDS^{s}
HIS-MBP-RIP2CARD
MBP
c
kDa
D_{300}
HIS-SUMO-NOD2CARDS^{s}
NOD2CARDS^{s}
HIS-MBP
RIP2CARD
d NOD2CARDS^{s} - RIP2CARD
e NOD2CARDS^{s} - RIP2CARD
f
kDa
D_{300}
HIS-MBP
RIP2CARD
g RIP2CARD
h RIP2CARD

Supplementary Figure 7: Purification and imaging of RIP2CARD and NOD2CARDS^{S}-RIP2CARD filaments

a: Domain organization of NOD2CARDS and RIP2CARD constructs used to produce filaments for cryo-EM application.

b: Size exclusion chromatography profile and corresponding 12.5% SDS-PAGE of NOD2CARDS^{S} -RIP2CARD sample before tag cleavage (VV= size exclusion chromatography void volume). The central peak fractions were used to recombine the tagged NOD2CARDS^{S} -RIP2CARD complex with the monomeric HIS-MBP-RIP2CARD.

c: 17% SDS-PAGE of NOD2CARDS^{S} -RIP2CARD filament sample used for cryo-EM (D_{300}=sample after dialysis with 300 kDa membrane cut off). Lanes in between the markers and D_{300} were deleted for clarity.

d-e: (d) Negative-stain and (e) cryo-EM images of RIP2CARD filaments with NOD2CARDS^{S} bound. Scale bars are 200 and 100 nm respectively.

f: 17% SDS-PAGE of RIP2CARD filament sample used for cryo-EM (D_{300}=sample after dialysis with 300 kDa membrane cut off).

g-h: (g) Negative-stain and (h) cryo-EM images of RIP2CARD filaments. Scale bars are 500 and 100 nm respectively.

a

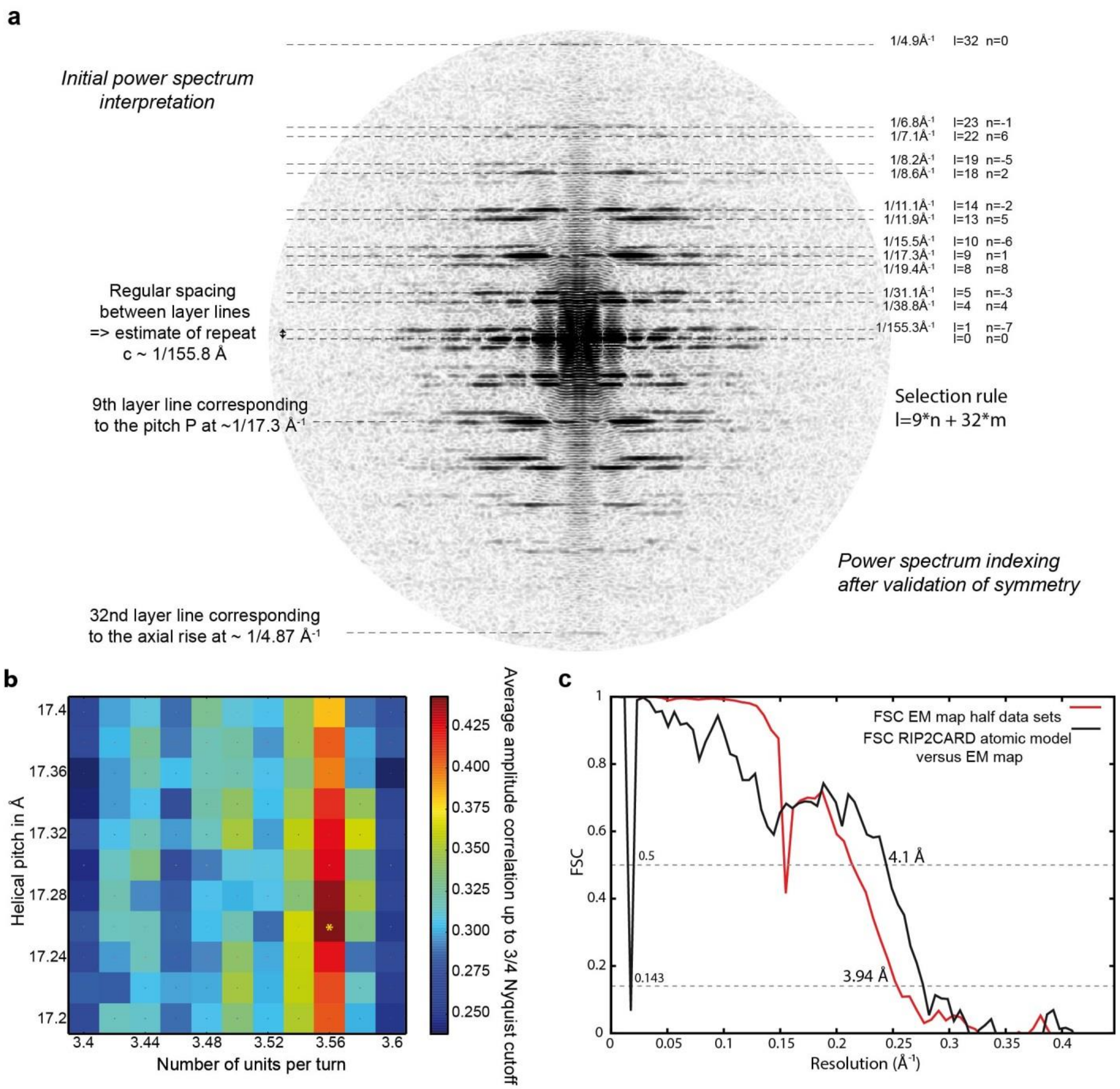

d

Image processing statistics

	Manual picked dataset used for 2D classification	Automatically picked dataset used for 3D reconstruction
Resolution at FSC 0.5 / 0.143, Å	-	4.5 / 3.94
Total length of non-overlapping segments before selection, µm	97	260
Number of filament sections before selection	4,125	4,443
Segment size, Å	508.2	400
Segment step size, Å	25	70
Number of segments before selection	23,742	37,043
Number of segments finally included in reconstruction	-	9,661
Pitch in Å / Units per turn [helical rise, Å / rotation, °]	-	17.26 / 3.56 [4.848 / -101.124]
Number of asymmetric units included in final reconstruction	-	135,254

Supplementary Figure 8: Symmetry determination and image processing statistics

a: Power spectra of a selected 2D class-average (Figure 6b), its initial interpretation (left) and the indexing (right) corresponding to the final symmetry (pitch 17.26 Å, 3.56 units/turn).

b: Symmetry refinement. The maximum correlation (indicated by an asterisk) occurs at units per turn/pitch of 3.56/17.26 Å.

c: Fourier Shell Correlation (FSC) curve between half data sets (red) and FSC curve between unfiltered, unsharpened map and the final atomic model of RIP2CARD within the filament.

d: Image processing statistics of the datasets used for 2D classification and 3D reconstruction.

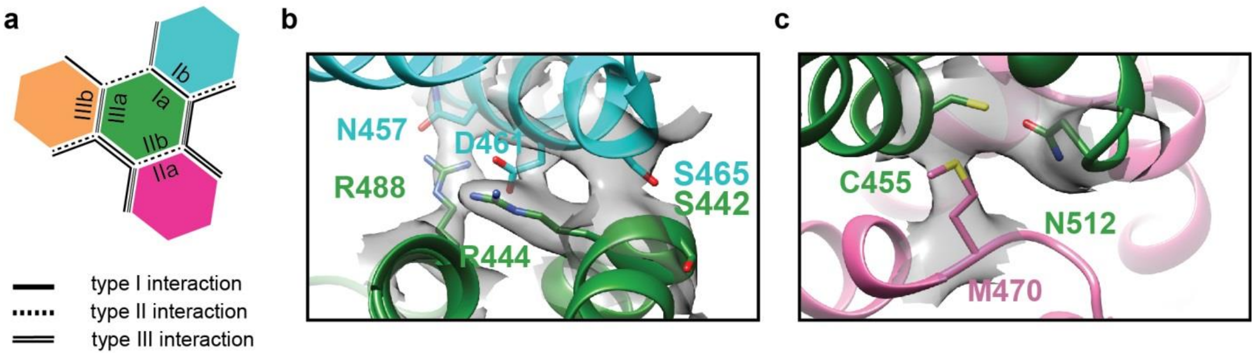

Supplementary Figure 9: Cryo-EM density at type I and type II interfaces

a: Relative orientations of type I, type II and type III interface within RIP2CARD filament.

b-c: EM density at (b) type I and (c) type II interfaces. Corresponding residue interactions are described in the main text.

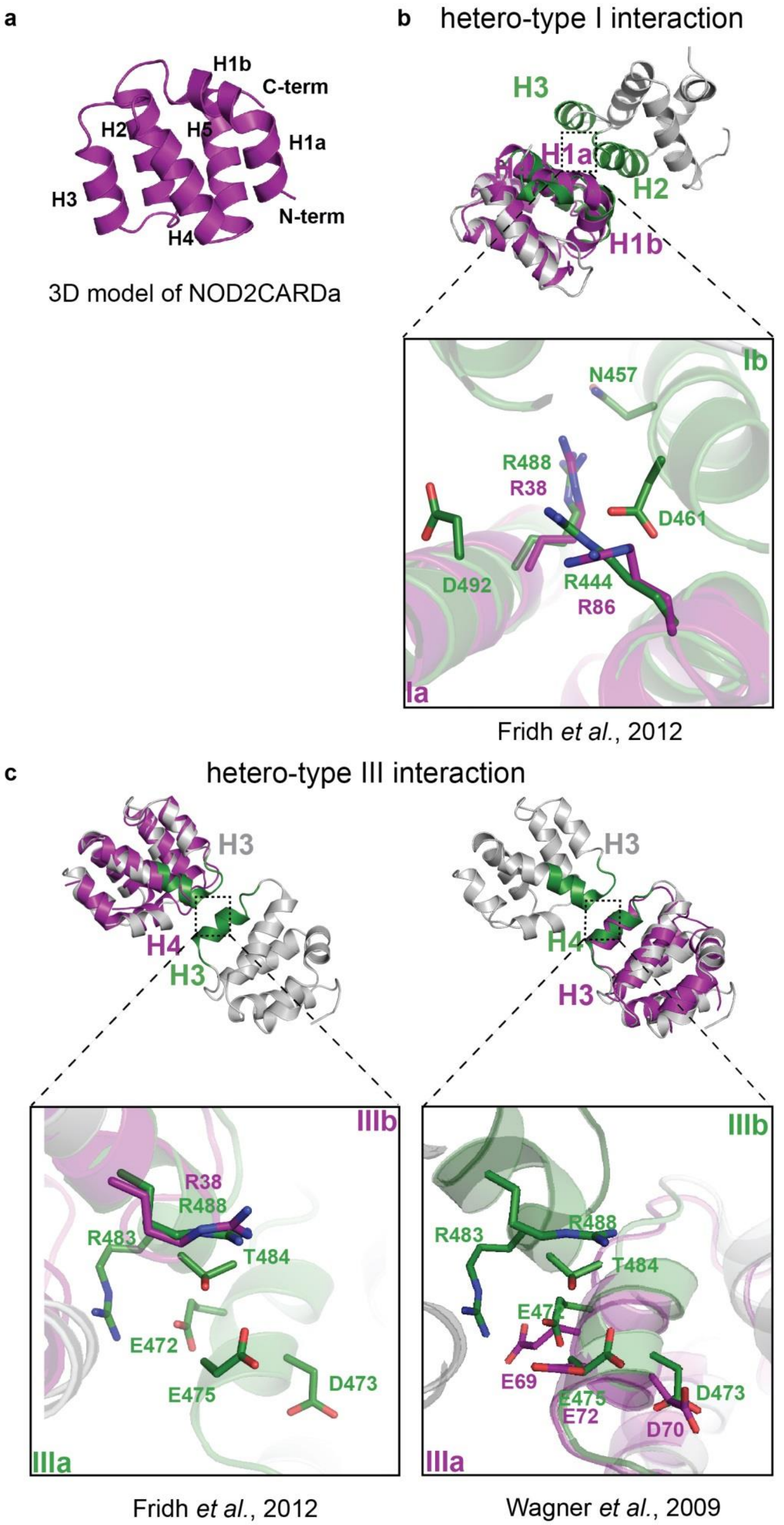
a
H1b
C-term
H2
H5
H1a
H3
H4
N-term
3D model of NOD2CARDa
b
hetero-type I interaction
H3
H1a
H2
H1b
Ib
N457
R488
R38
D461
D492
R444
R86
Ia
Fridh et al., 2012
c
hetero-type III interaction
H3
H4
H3
H3
H4
H3
IIIb
R38
R488
R483
T484
E472
E475
D473
IIIa
IIIb
R483
R488
T484
E69
E475
E72
D473
D70
IIIa
Fridh et al., 2012
Wagner et al., 2009

Supplementary Figure 10: Model of the hetero-CARD type I and III interactions

a: Ribbon diagram of NOD2CARDa model produced using Swiss-Model[3].

b-c: Ribbon diagrams of the putative hetero-CARD dimer interactions at (b) type I and (c) type III interfaces. The NOD2CARDa model was superposed on one of the RIP2CARD molecules in the equivalent RIP2CARD homo-dimers. The insets show the putative residue interactions at each interface, green (RIP2), purple (NOD2). The NOD2CARDa residues shown have been implicated in interactions with RIP2CARD[4,5]. In the hetero type I interface, NOD2CARDa type Ia surface comprises residues R38 and R86, which are equivalent to R488 and R444 in RIP2CARD, and therefore might preserve the polar interactions with N547 and D461 in RIP2CARD type Ib surface. For the hetero-CARD type III interaction, the experimental data is consistent with either basic residues of NOD2 interacting with the acidic patch on RIP2 (left) or vice versa (right).

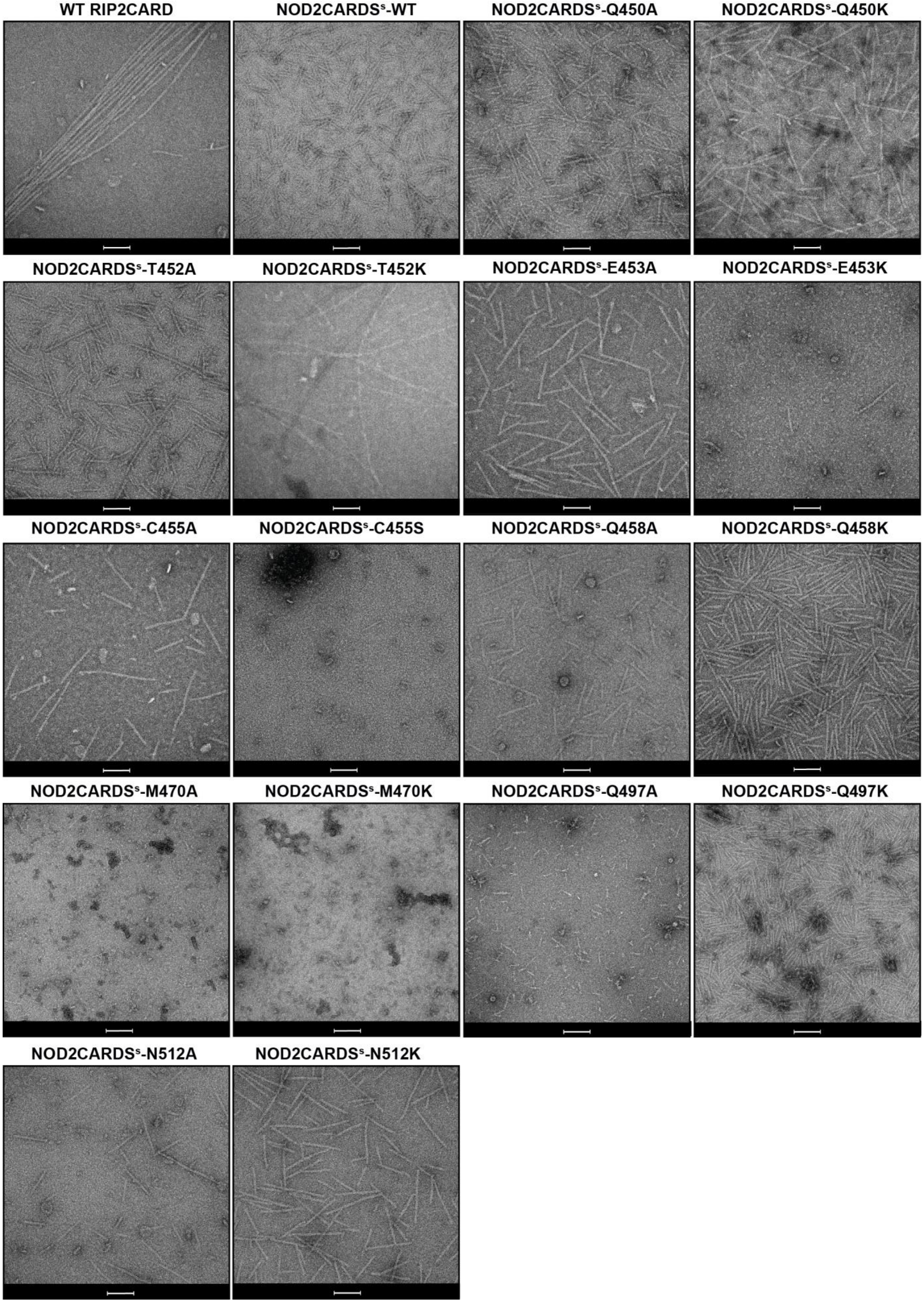
WT RIP2CARD
NOD2CARDS^{s}-WT
NOD2CARDS^{s}-Q450A
NOD2CARDS^{s}-Q450K
NOD2CARDS^{s}-T452A
NOD2CARDS^{s}-T452K
NOD2CARDS^{s}-E453A
NOD2CARDS^{s}-E453K
NOD2CARDS^{s}-C455A
NOD2CARDS^{s}-C455S
NOD2CARDS^{s}-Q458A
NOD2CARDS^{s}-Q458K
NOD2CARDS^{s}-M470A
NOD2CARDS^{s}-M470K
NOD2CARDS^{s}-Q497A
NOD2CARDS^{s}-Q497K
NOD2CARDS^{s}-N512A
NOD2CARDS^{s}-N512K

Supplementary Figure 11: Effect of type II interface residue mutations on RIP2CARD polymerization *in vitro*.

Example negative-stain electron micrographs of wild-type RIP2CARD and NOD2CARDS^{S}-RIP2CARD filaments (top left pair) and NOD2CARDS^{S} combined with all RIP2CARD mutants, after tag cleavage. Scale bars are 100 nm.

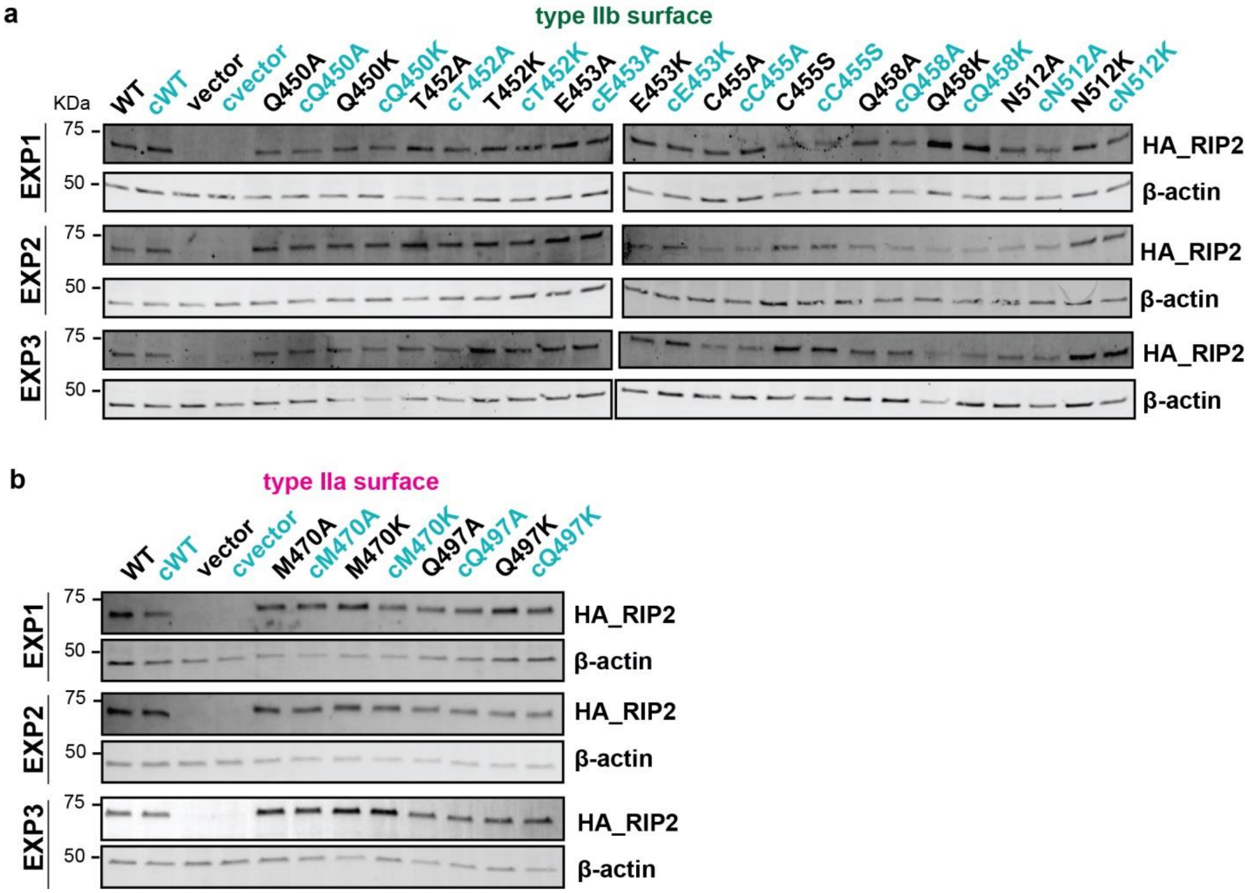

Supplementary Figure 12: Western blot for in-cell based assay.

One Western blot per biological replicate for (a) type IIb and (b) type IIa surface mutants is shown. Replicates are labelled as: EXP1, EXP2, EXP3 (EXP=experiment). For each EXP, expression of wild-type (WT) HA-RIP2fl and mutants (right) and β-actin (left) are shown. For each construct, the results for cells induced by either MDP or its control (cMDP, c) are labelled in black and cyan respectively.

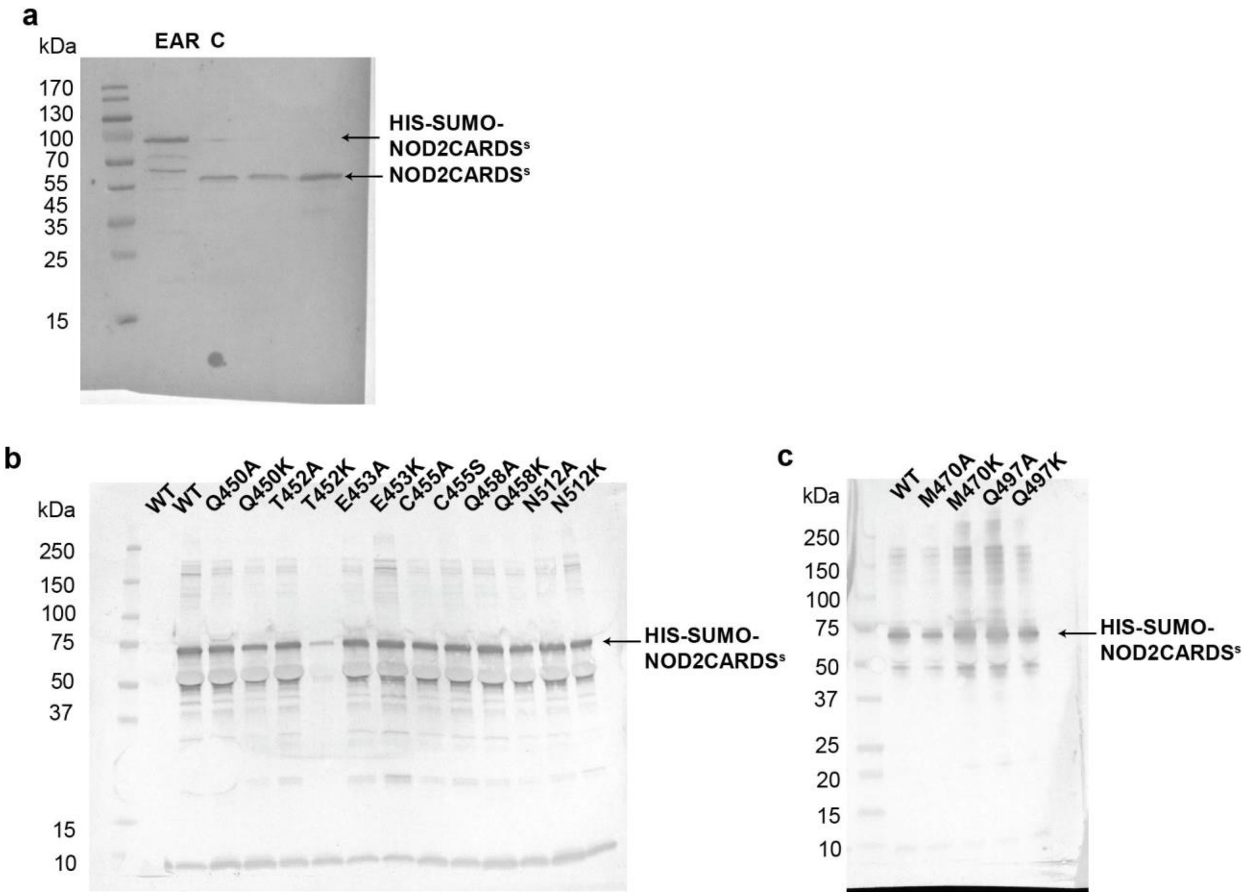

Supplementary Figure 13: Uncropped blots corresponding to (a) Figure 5c, (b-c) Figure 8b.

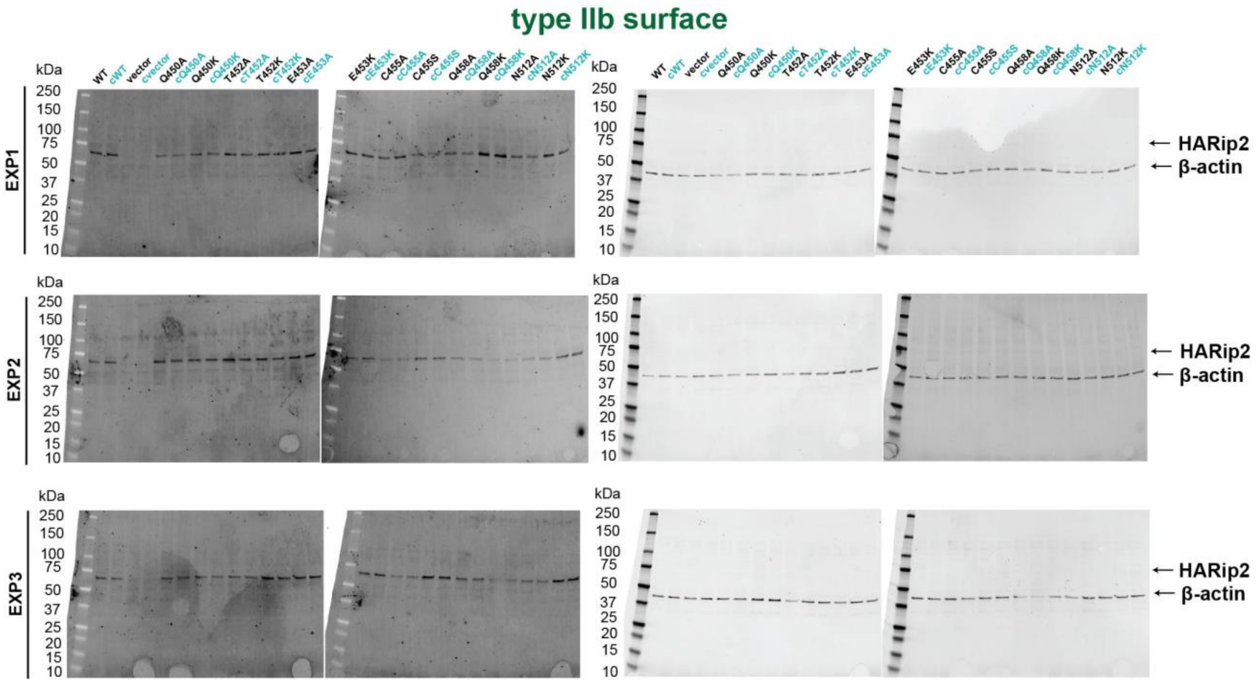

Supplementary Figure 14: Uncropped blots corresponding to Figure 9b and Supplementary Figure 12a.

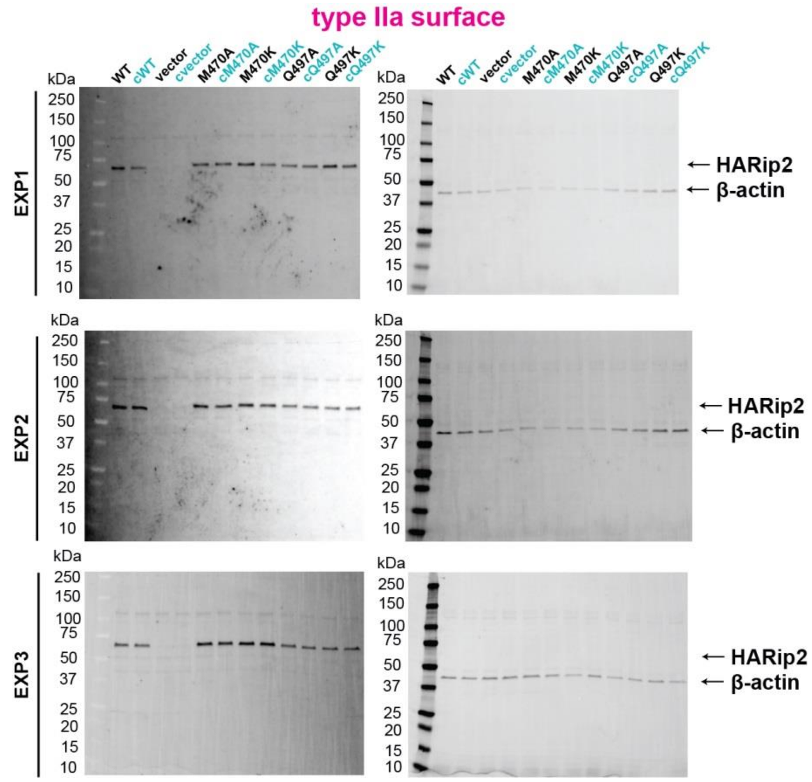

Supplementary Figure 15: Uncropped blots corresponding to Figure 9b and Supplementary Figure 12b.

Supplementary Table 1. Crystallographic data collection and refinement statistics.

	crystMBP-RIP2CARD (PDB 6GFJ)
**Data collection**	
Space group	*P*21
Cell dimensions	
a, *b*, *c* (Å)	89.46,121.57,123.16
α, β, γ (°)	90.00, 109.04, 90.00
Resolution (Å)	46.98-3.29(3.38-3.29)*
R_{meas}	0.19 (1.37)
$I / \sigma I$	5.45 (0.99)
CC(1/2)	0.993 (0.437)
Completeness (%)	98.5 (86.9)
Redundancy	3.80 (3.42)
**Refinement**	
Resolution (Å)	46.98-3.30
No. reflections	37320
R_{work} / R_{free}	0.235/0.266
No. atoms	14268
Protein	2841, 712 (MBP_Chain A, CARD chain S)
	2841, 712 (MBP_Chain B, CARD chain T)
	2841, 703 (MBP_Chain C, CARD chain X)
	2841, 712 (MBP_Chain D, CARD chain Y)
Ligand/ion	4 maltose (MAL)
Water	0
B-factors (Å^2)	131.50
Protein	124.40, 132.33 (MBP_Chain A, CARD chain S)
	133.69, 132.33 (MBP_Chain B, CARD chain T)
	127.31, 152.31 (MBP_Chain C, CARD chain X)
	132.25, 146.02 (MBP_Chain D, CARD chain Y)
Ligand/ion	95.47 (MAL_A)
	106.11 (MAL_B)
	101.07 (MAL_C)
	146.02 (MAL_D)
R.m.s. deviations	
Bond lengths (Å)	0.007
Bond angles (°)	1.053

Values in parentheses are for the highest-resolution shell.

Supplementary Table 2: Solid state NMR chemical shift assignment of RIP2CARD on the basis of ^{1}H-detected experiments

	H	N	C	Cα	Cβ
441Q	7.58	117.10	175.76	56.01	24.91
442S	8.07	113.32	172.39	57.96	60.35
443K	7.46	119.72	173.10	50.53	27.41
444R	7.20	122.69	173.26	57.73	27.87
445E	8.06	112.45	174.23	56.43	27.27
446D	7.21	116.65	174.59	53.58	38.23
447I	8.50	120.33	174.81	63.27	34.72
451M			173.00		
452T	9.02	118.80	171.85	58.46	67.89
453E	9.31	120.24	174.60	57.30	26.43
454A	8.60	116.91	176.20	51.86	15.96
455C	7.51	122.61	173.81	59.55	
456L	8.29	125.61	174.99	55.24	37.21
457N	8.60	118.37	174.17	52.82	34.50
458Q	8.66	119.75	175.62	55.98	26.91
459S	8.20	117.02	172.14	60.85	59.66
460L	7.87	120.66	175.10	55.23	38.13
461D	8.66	118.57	175.24	55.14	37.94
462A	8.16	123.45	176.99	51.57	15.97
463L	8.06	120.01	176.54	54.94	39.19
464L	8.72	121.76	178.06	54.85	39.48
465S	8.36	117.19	172.42	58.31	60.94
466R	7.04	117.75	171.32	52.16	28.06
467D	8.11	118.96	172.25	52.12	37.09
468L	8.34	114.84	172.33	52.18	41.59
469I	6.80	114.12	169.77	53.79	39.20
470M	9.86	127.86	174.96	53.24	30.43
471K	8.92	125.45	175.29	57.41	26.87
472E	9.39	117.53	174.58	56.32	25.65
473D	6.66	118.12	175.75	54.98	37.66
474Y	8.33	123.02	174.40	57.63	34.02
475E	8.89	123.35	176.15	56.17	26.51
476L	8.39	120.42	176.00	54.49	39.75
477V	7.44	116.70	173.40	63.45	28.80
478S	7.88	109.32	172.00	58.27	60.95
479T	7.50	107.11	172.58	58.26	66.98
480K	7.07	123.88	173.14	50.56	26.44
481P			174.05	62.04	29.59
482T	6.83	104.25	170.07	54.76	69.71
483R	8.91	124.65	174.57	57.18	25.62
484T	9.06	113.77	172.25	65.27	66.64
485S	8.08	114.26	175.54	58.45	60.30
486K	8.48	126.21	175.06	57.51	31.50
487V	8.28	118.82	175.24	63.65	28.48
488R	8.76	118.43	174.21	58.27	26.87
489Q	8.13	118.26	176.17	55.07	24.02
490L	8.60	124.50	176.92	55.38	37.51
491L	8.94	123.70	176.50	55.35	36.03
492D	9.22	123.48	176.19	54.24	36.46
493T	8.01	117.34	171.83	63.90	65.84
494T	8.26	120.35	171.54	63.97	65.50
495D	7.40	117.43	175.53	53.74	39.08
496I	6.81	115.09		59.30	
498G	7.88	104.37	171.21	42.29	
499E	8.36	120.74	174.87	55.88	27.42
500E	8.71	118.19	175.15	56.58	25.78
501F	7.75	120.48	173.76	56.66	37.57
502A	8.05	118.45	175.88	52.14	15.36
503K	8.81	116.74	175.61	57.42	29.70
504V	7.19	118.53	174.10	63.07	28.48
505I	6.96	118.30	174.64	61.56	34.52
506V	8.18	117.27	173.77	64.36	28.25
507Q	7.92	117.47	174.61	55.74	25.07

Supplementary Table 3. Cryo-EM data collection, refinement and validation statistics

	RIP2CARD filament (PDB-6GGS, EMD-4399)
**Data collection and processing**	
Magnification	41270x
Voltage (kV)	300
Electron exposure (e–/Å^2)	50
Defocus range (μm)	-1.5 to 3
Pixel size (Å)	1.21
Symmetry imposed	Helical
Initial particle images (no.)	37043
Final particle images (no.)	9661
Map resolution (Å)	3.94
FSC threshold	0.143
Map resolution range (Å)	3.6-4.2
**Refinement**	
Initial model used (PDB code)	6GFJ (Chain S), this study
Model resolution (Å)	3.3
Map sharpening *B* factor (Å^2)	-200
Model composition	Chain F
Protein residues	85 (residues 433-518)
B factors (Å^2)	124.7
R.m.s. deviations	
Bond lengths (Å)	0.0063
Bond angles (°)	1.43
Validation	
MolProbity score	2.08
Clashscore	14.46
Poor rotamers (%)	1.27
Ramachandran plot	
Favored (%)	95.24
Allowed (%)	4.76
Disallowed (%)	0

Supplementary Table 4: Helical parameters of DD filament structures

Filament	Rotation per subunit (^{0})	Rise per subunit (Å)	Number of molecules per turn	Resolution (Å)	Reference
RIP2CARD	-101.12	4.84	3.56	4.0	this paper
Caspase-1	-100.21	5.1	3.59	4.8	Lu, A. *et al* 2016
Bl10	-100.8	5	3.57	4	David, L. *et al* 2018
MAVS	-101.1<br>-101.21	5.1<br>5.06	3.56<br>3.56	3.6/4.12	Wu, B. *et al* 2014<br>Xu, L. *et al* 2014

Supplementary Table 5: Oligos used for mutagenesis

Constructs	Oligos
**Q450A**	5′-GACATTGTGAACGCAATGACAGAAGCC -3′
**Q450K**	5′-AAGACATTGTGAACAAAATGACAGAAGCC-3′
**T452A**	5′-GTGAACCAAATGGCAGAAGCCTGC-3′
**T452K**	5′-GTGAACCAAATGAAAGAAGCCTGC-3′
**E453A**	5′-CCAAATGACAGCAGCCTGCCTTAAC-3′
**E453K**	5′-CCAAATGACAAAAGCCTGCC-3′
**C455A**	5′-GACAGAAGCCGCCCTTAACCAGTC-3′
**C455S**	5’-GCCCTTCTGTGCAGGGA-3′
**Q458A**	5′-CCTGCCTTAACGCGTCGCTAGATG-3′
**Q458K**	5′-GCCTTAACAAGTCGCTAGATGCC-3′
**M470A**	5’-GGACTTGATCGCGAAAGAGGACTATG-3’
**M470K**	5’-GGACTTGATCAAGAAAGAGGACTATG-3’
**Q497A**	5’-CTACTGACATCGCAGGAGAAGAATTTG-3’
**Q497K**	5’-CTACTGACATCAAAGGAGAAGAATTTG-3’
**N512A**	5′-GTACAAAAATTGAAAGATGCAAAACAAATGGGTCTTCAG-3′
**N512K**	5′-GTACAAAAATTGAAAGATAAAAAACAAATGGGTCTTCAG-3′
**pcDNA-HA-RIP2**	5′-TGCAGACCCTGCAGACCCAGCGTAGTCTGGGACGTCGTA TGGGTACTCGAGGGCCATGGTGGTATC-3'
**pcDNA-HA-RIP2**	5'-GCAGGGTCTGCAATGAACGGGGAGGCCATCTGCAGCGC CCTGCCCACCATTCCCTACCACAAACTC-3'

Supplementary References.

1 Poirot, O., Suhre, K., Abergel, C., O'Toole, E. & Notredame, C. 3DCoffee@igs: a web server for combining sequences and structures into a multiple sequence alignment. *Nucleic acids research* **32**, W37-40, doi:10.1093/nar/gkh382 (2004).

2 Robert, X. & Gouet, P. Deciphering key features in protein structures with the new ENDscript server. *Nucleic acids research* **42**, W320-324, doi:10.1093/nar/gku316 (2014).

3 Biasini, M. *et al.* SWISS-MODEL: modelling protein tertiary and quaternary structure using evolutionary information. *Nucleic acids research* **42**, W252-258, doi:10.1093/nar/gku340 (2014).

4 Wagner, R. N., Proell, M., Kufer, T. A. & Schwarzenbacher, R. Evaluation of Nod-like receptor (NLR) effector domain interactions. *PloS one* **4**, e4931, doi:10.1371/journal.pone.0004931 (2009).

5 Fridh, V. & Rittinger, K. The tandem CARDs of NOD2: intramolecular interactions and recognition of RIP2. *PloS one* **7**, e34375, doi:10.1371/journal.pone.0034375 (2012).
